# The permanently chaperone-active small heat shock protein Hsp17 from *Caenorhabditis elegans* exhibits topological separation of its N-terminal regions

**DOI:** 10.1016/j.jbc.2022.102753

**Published:** 2022-11-26

**Authors:** Annika Strauch, Benjamin Rossa, Fabian Köhler, Simon Haeussler, Moritz Mühlhofer, Florian Rührnößl, Caroline Körösy, Yevheniia Bushman, Barbara Conradt, Martin Haslbeck, Sevil Weinkauf, Johannes Buchner

**Affiliations:** 1 Center for Protein Assemblies and Department of Chemistry, Technische Universität München, Garching, Germany; 2 Faculty of Biology, Ludwig-Maximilians-University Munich, Planegg-Martinsried, Germany; 3 Debye Institute for Nanomaterials Science, Utrecht University, Utrecht, Netherlands

**Keywords:** molecular chaperone, small heat shock protein, protein structure, protein aggregation, Hsp-17, Hsp17, sHsps, ACD, α-crystallin domain, CD, Circular Dichroism, CS, citrate synthase, CTR, C-terminal region, FA, formic acid, HDX-MS, hydrogen-deuterium exchange mass spectrometry, IAA, iodoacetamide, MDH, malate dehydrogenase, NTR, N-terminal region, RT, room temperature, sHsps, small Heat shock proteins

## Abstract

Small Heat shock proteins (sHsps) are a family of molecular chaperones that bind nonnative proteins in an ATP-independent manner. *Caenorhabditis elegans* encodes 16 different sHsps, among them Hsp17, which is evolutionarily distinct from other sHsps in the nematode. The structure and mechanism of Hsp17 and how these may differ from other sHsps remain unclear. Here, we find that Hsp17 has a distinct expression pattern, structural organization, and chaperone function. Consistent with its presence under nonstress conditions, and in contrast to many other sHsps, we determined that Hsp17 is a mono-disperse, permanently active chaperone *in vitro,* which interacts with hundreds of different *C. elegans* proteins under physiological conditions. Additionally, our cryo-EM structure of Hsp17 reveals that in the 24-mer complex, 12 N-terminal regions are involved in its chaperone function. These flexible regions are located on the outside of the spherical oligomer, whereas the other 12 N-terminal regions are engaged in stabilizing interactions in its interior. This allows the same region in Hsp17 to perform different functions depending on the topological context. Taken together, our results reveal structural and functional features that further define the structural basis of permanently active sHsps.

Small heat shock proteins (sHsps) are ATP-independent molecular chaperones present in all three domains of life (1) as well as in viruses like cyanophages (2). The number of sHsps varies between species. For instance, in humans, there are 10 different sHsps present (3, 4) and plant genomes usually encode between 19 to 36 sHsps (5, 6). In comparison, prokaryotes contain mostly only one or two sHsp genes (1, 7, 8). In the nematode *Caenorhabditis elegans*, 16 different sHsps have been reported (9). It is still enigmatic why the sHsp family expanded so strongly in multicellular organisms.

The primary structure of sHsps shows a conserved organization with three functional segments: the α-crystallin domain (ACD) which is surrounded by the highly flexible N-terminal region (NTR) and a short C-terminal region (CTR) (10, 11, 12, 13). The ACD is the signature domain of sHsps and shows the highest sequence identity within the protein superfamily. It exhibits a β-sandwich structure similar to the immunoglobulin fold (14).

One of the fascinating features of sHsps is their ability to form ensembles of large oligomers, mostly in the range of 12 to 40 monomers, with dimers as the basic building block (14, 15, 16, 17). The structures of the oligomers are dynamic, which appears to enable complex formation with unfolding proteins (12), rescuing them from irreversible aggregation *in vitro* (18, 19). Also, coaggregation of sHsps with substrate proteins has been observed (20, 21). As the function of sHsps is ATP-independent, they can be seen as holdases, which bind nonnative proteins efficiently and cooperate with ATP-dependent foldases such as Hsp70 for refolding (5, 12, 13, 15, 22, 23). sHsps are not only required for protection against severe, external stress conditions, they also play an important role in processes like development (24), differentiation (25), regulation of angiogenesis (26), apoptosis (27, 28, 29), tumorigenesis (30), as well as in the proteasomal degradation of proteins (31).

Despite the insights into the structure and function of sHsps in recent years, important questions remain. Especially, it is still enigmatic which features discriminate the different sHsp family members within one multicellular organism. They could be functionally distinct or deviate in their availability when expressed only at certain stages of development or under specific conditions.

In *C. elegans*, 8 out of the 16 different sHsps are classified into two main groups, the Hsp12 (4 members) and Hsp16 families (4 members) (32, 33). Expression of the Hsp16 family members is stress-induced (32), whereas the sHsps F08H9.3 and F08H9.4 are expressed constitutively and Sip1 is mainly expressed in embryos (24, 34). The tissue distribution is also different for members of the Hsp12 family, which localize to the cytoplasm of sperm cells as well as vulva muscles (35) and the Hsp16 family, which is found in the intestine, pharynx, hypodermis, as well as body muscle (36, 37, 38).

Hsp17 (or Hsp-17 according to the *C. elegans* nomenclature) is set apart from the sHsps mentioned above by its isolated position in the phylogenetic tree of *C. elegans* sHsps. It was previously shown to be expressed under physiological conditions in different tissues of young adult worms (39) and its expression level was only induced slightly in response to heat stress (40). Knockdown of Hsp17 by RNAi led to a slight reduction of the lifespan, reduced offspring, and reduced survival after heat treatment. Functionally, it was shown to interact with unfolding proteins and affect their aggregation (39). Interestingly, Hsp17 orthologs in parasitic worms like *Strongyloides ratti* and *stercoralis* seem to be important to adapt to the host environment during infection (41, 42, 43).

These findings suggested that Hsp17 plays a special role in *C. elegans* proteostasis. However, the current knowledge on its structure and function did not yet allow explaining its characteristic traits and the underlying molecular mechanism of its chaperone activity. Here, we set out to establish the chaperone mechanism of Hsp17 as well as the structure of the oligomeric complex by cryo-EM.

## Results

### Hsp17 is uniquely expressed throughout the life cycle and is phylogenetically separated from other *C. elegans* sHsps

Hsp17 has been shown to be expressed in the pharynx, excretory canal, intestine, and pseudocoelom (39) in young adult worms. To determine the expression pattern of Hsp17 during development, we injected worms with a fosmid encoding a Hsp17::GFP fusion protein under the endogenous promoter. We observed Hsp17::GFP expression already in the embryonic stage, where it was detected in the excretory cells (Fig. S1, *C*–*E*; white arrows). After hatching, Hsp17 was additionally expressed in the pharynx and at later stages, in the intestine and around the anus (Fig. S1*A*, F-M). In adult worms, a similar expression pattern was observed consistent with the literature (39) (Fig. S1*A* and N-R). In male worms (Fig. S1*B* and S-U), Hsp17 was additionally present in cells around the tail (Fig. S1*B*, S and U; black arrows). In summary, we conclude that Hsp17 is expressed in an increasing number of tissues during development to adult worms. The expression pattern suggests specific, constitutive functions of Hsp17 which might be distinct from other *C. elegans* sHsps.

**Figure 1:**
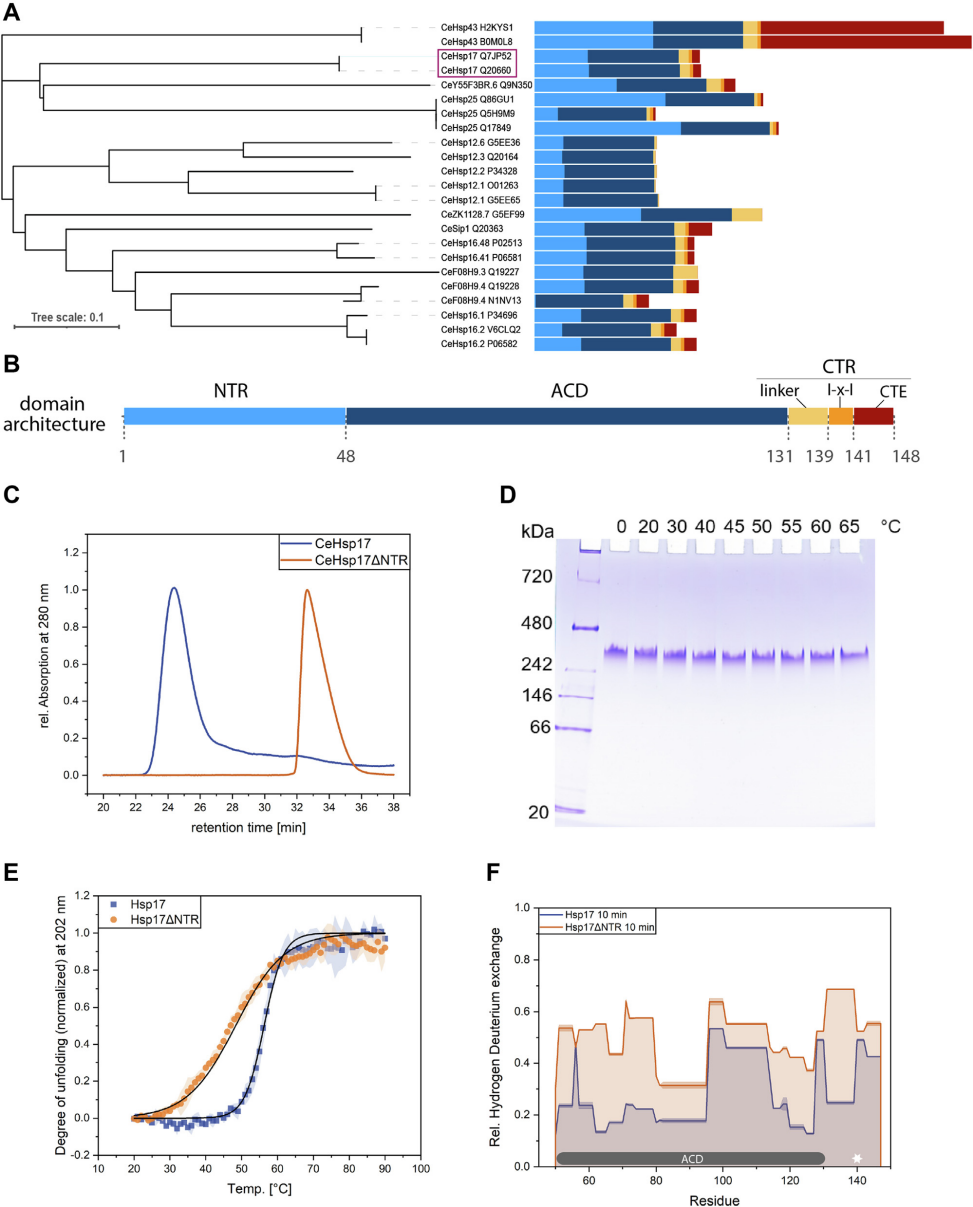
**Phylogeny, structure, and oligomerization of Hsp17.***A*, phylogenetic analysis of the *Caenorhabditis elegans* sHsps and their splice variants. Domain architecture was determined by alignment with Sip1. It is visualized in different colors with the same assignment as in (*B*) where a more detailed view of Hsp17 is shown. *C*, Size exclusion chromatography-HPLC measurements of Hsp17 and Hsp17ΔNTR (n = 3). *D*, native PAGE of heat-treated Hsp17 (n = 3). *E*, thermal transition of Hsp17 and Hsp17ΔNTR (n = 3; shades show SD). *F*, comparison of HD exchange between Hsp17 and Hsp17ΔNTR. The two proteins were superimposed by their ACD, the I-x-I motif is indicated with a *white star*, and the peptide coverage is shown in Fig. S4 (n = 3 technical replicates; measured twice; error bars show SD). sHsps, small Heat shock proteins; ACD, α-crystallin domain.

To further define the role of Hsp17, we analyzed its phylogenetic relationship within the sHsp family of *C. elegans*. We find that Hsp17 is evolutionary distinct from the Hsp12 and Hsp16 families (Fig. 1*A*). We found the highest sequence identity (33%) between Hsp17 and Hsp25; for other *C. elegans* sHsps, it ranges from 19.7 to 31.9% (Fig. S2). Structurally, Hsp17 follows the general scheme observed for sHsps: it contains an ACD domain of average length, an N-terminal region, and a C-terminal tail (Fig. 1*B*). Thus, Hsp17 differs on the sequence level from other *C. elegans* sHsps, while the general organization of the domains and their length is conserved.

### Hsp17 forms stable oligomers of 24 subunits defined by the NTRs

An important question in the context of sHsp structure and function is whether dynamic ensembles of different-sized oligomers are formed. To test this for Hsp17, the quaternary structure of Hsp17 and of a variant lacking the N-terminal region (Hsp17ΔNTR) was analyzed by size exclusion chromatography (SEC)-HPLC, negative stain EM, and native PAGE (Figs. 1, *C* and *D* and S3, *A*–*E*). Hsp17 forms large oligomeric structures with a size of 418 kDa which corresponds to a 24-mer (theoretical Mw 418,032 Da) (Figs. 1*C* and S3*A*, *B* and *D*), consistent with previous results (39, 44). Additionally, the tailing peak of the full-length protein could indicate the presence of dimers involved in subunit exchange (45, 46). Hsp17ΔNTR exhibits a SEC-peak around 38 kDa (corresponding to 3.5 monomers) (Figs. 1*C* and S3, *C* and *D*), suggesting a mixture of dimers and tetramers. Despite that, it should be noted that the NTR-truncated variant has an apparent higher molecular mass in the native PAGE (Fig. S3*E*). Thus, the NTR plays an important role in oligomerization. Importantly, negative stain EM revealed that different from many other sHsps, Hsp17 complexes are mono-disperse (Fig. S3, *A* and *B*).

Furthermore, in contrast to results obtained for a His-tagged variant of Hsp17, which forms supermolecular assemblies after incubation at 50 °C (44), we could not detect changes in quaternary structure for heat-treated Hsp17 and Hsp17ΔNTR using native PAGE or negative stain EM. Also, no indication for the disassembly of the Hsp17 oligomer was observed at elevated temperatures (Figs. 1*D*, 5, S8 and S9) as it is the case for other sHsps such as Hsp26 from yeast (47). Thus, Hsp17 is a stable 24-mer that does not readily dissociate. This raises question about its mechanism of action as typically, dissociation into smaller active oligomers is assumed to be a prerequisite for substrate binding. Beyond that, it cannot be excluded that Hsp17 changes its oligomeric distribution under certain *in vivo* conditions, for example, posttranslational modifications, which have been undetected up to now.

The thermal stability of Hsp17 and Hsp17ΔNTR as monitored by Circular Dichroism (CD) spectroscopy (Fig. 1*E*) revealed melting temperatures of 56.4 °C for Hsp17 and 47.9 °C for Hsp17ΔNTR. The difference of 8.5 °C shows that the NTR contributes significantly to the thermal stability of Hsp17 suggesting interactions in the oligomeric structure. To obtain further insight into the structural dynamics of Hsp17 and Hsp17ΔNTR, we performed hydrogen-deuterium exchange mass spectrometry (HDX-MS). Comparing Hsp17 and Hsp17ΔNTR, based on an identical peptide coverage map, an overall increase in the HD exchange rates becomes apparent for the truncated variant. This is consistent with the formation of smaller oligomeric species by Hsp17ΔNTR, leading to a loss of contact sites. For the I-x-I motif (position 138–140) in the CTR, the HD exchange rates of full-length protein and the truncation variant is similar, demonstrating that this motif is engaged in similar interactions in Hsp17 and Hsp17ΔNTR (Figs. 1*F* and S4, *A*–*C*). Moreover, analysis of the Hsp17 HDX-MS measurement revealed a high HD exchange for the first nine N-terminal aa and the last 8 aa (Fig. S4, *D* and *E*).

Taken together, the picture revealed in the above analyses is that Hsp17 is a defined 24mer in which NTR-contacts play a major role in organizing the oligomer as deduced from the size of Hsp17ΔNTR, the changes in the HDX pattern, and the difference in stability compared to the full-length protein.

### The substrate spectrum of Hsp17 differs from that of other *C. elegans* sHsps

Besides tissue- and development-specific expression, differences in the substrate specificity may be the reason for the large number of different sHsps in *C. elegans*. To address this question, we determined the substrate spectra of Hsp17, Sip1, and Hsp16.2 in comparison. Co-immunoprecipitations (Co-IPs) were performed at a physiological (20 °C) and a stress temperature (37 °C) (48).

**Figure 2:**
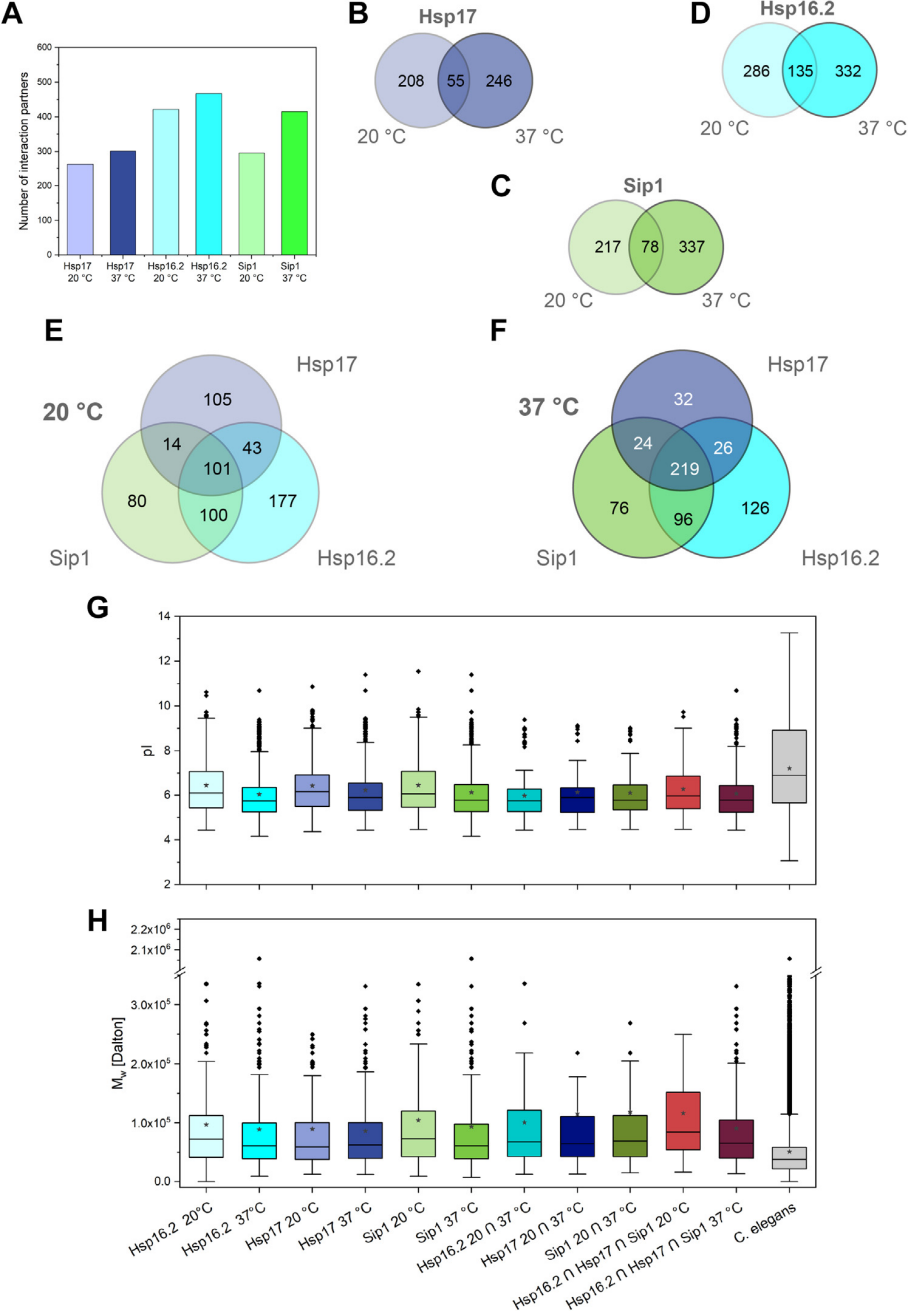
**Interactome analysis of Hsp17 in relation to Hsp16.2 and Sip1.** Co-IP results shown as bar chart (*A*) and Venn diagrams (*B*-*F*) comparing interaction partners (log2fc ≥ 2, *p*-value ≤ 0.05) of Hsp17 (*B*), Hsp16.2 (*C*), and Sip1 (*D*) at 20 °C and 37 °C. Further comparison between all three sHsps at 20 °C (*E*) and 37 °C (*F*). Detailed analysis of the hits concerning their pI (*G*) and molecular weight (*H*). *Rhombuses* in the box plots indicate outliners and *stars* were used for the mean value (Co-IP samples: n ≥ 3). sHsps, small Heat shock proteins.

For all three sHsps tested, hundreds of interaction partners could be identified by mass spectrometry with the number of interactors increasing with temperature (Fig. 2*A*). Dependent on the sHsp, 20 to 32% of the proteins interacting at 20 °C were retrieved in the 37 °C samples (Fig. 2, *B*–*D*) indicating that the substrate spectrum is different under physiological and stress conditions. Interestingly, 40% of the interactors of Hsp17 were unique when compared to the Sip1 and Hsp16.2 interactome. At 37 °C, the overlap for common interactors increased from 101 to 219, while the unique ones stayed roughly constant (Fig. 2, *E* and *F*). This higher overlap at 37 °C might indicate that although differences in the activation mechanism of the sHsps investigated may exist, these seem to have only a minor impact on the stress interactomes. Thus, under stress conditions, the substrate spectra of the tested sHsps converge. Moreover, in agreement with their phylogenetic relationship (Fig. 1*A*), Hsp16.2 and Sip1 share more interaction partners than they do with Hsp17 (Figs. 2, *E* and *F* and S5*A*). To test for common properties of the interactomes, general protein parameters were determined and compared to the *C. elegans* proteome (Figs. 2, *G* and *H* and S5, *B* and *C*). This analysis revealed that, for the investigated sHsps, the median Mw of the interactome is higher and the median pI is lower than the median of the *C. elegans* proteome. For the Hsp17 interactome, we found that proteins with a basic pI are underrepresented compared to the bimodal distribution of the *C. elegans* proteome (Fig. S5*B*). The hydrophobicity of the interactors is in the medium range of the *C. elegans* proteome (Fig. S5, *C* and *D*). While it is not possible to conclude clear properties for the Hsp17 interactome, there is some specificity concerning more negative charges and higher molecular weight.

### The chaperone mode of Hsp17 depends on substrates and conditions

**Figure 3:**
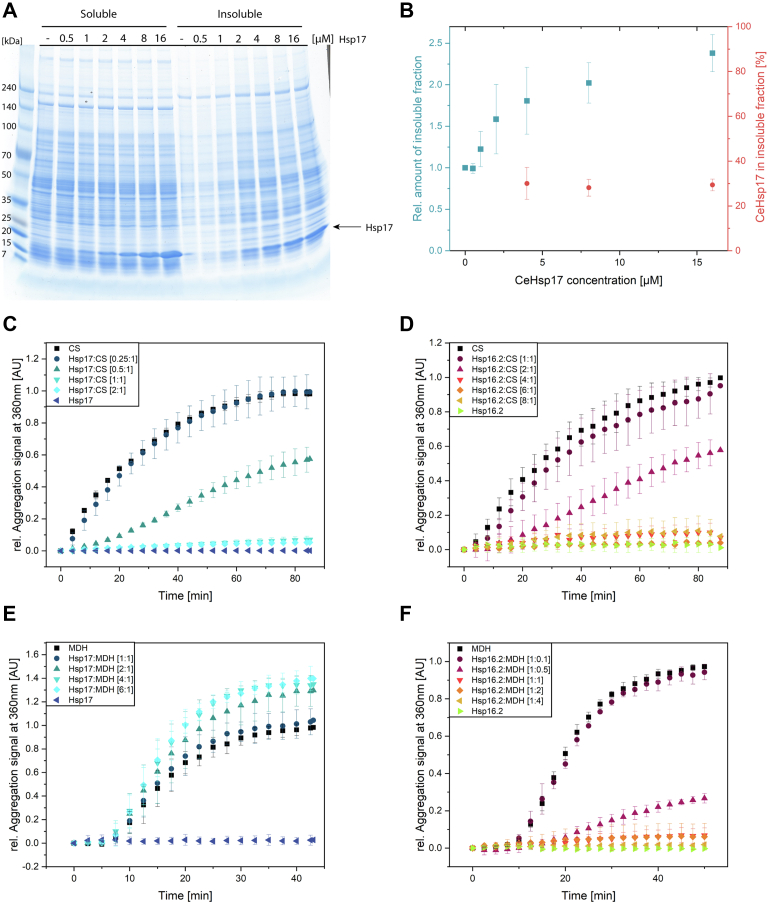
**Analysis of the substrate-dependent chaperone activity of Hsp17 and Hsp16.2.***A*, *in vitro* aggregation assay with *Caenorhabditis elegans* lysates, samples were separated into a soluble and insoluble fraction after coincubation with different amounts of Hsp17 (n = 3). *B*, quantification of the insoluble fraction of the lysate aggregation assay and percentage of the added Hsp17 in the insoluble fraction. Quantification was performed with ImageJ (n = 3; error bars show SD). Suppression of thermal-induced aggregation of CS by (*C*) Hsp17 and (*D*) Hsp16.2 which also suppresses the aggregation of the substrate (n = 3). Moreover, chaperone assays with MDH were performed with (*E*) Hsp17 and (*F*) Hsp16.2 (n = 2). Both sHsps were added at different molecular ratios. sHsps, small Heat shock proteins; MDH, malate dehydrogenase; CS, citrate synthase.

It is reasonable to assume that the specific contribution of Hsp17 to proteostasis in *C. elegans* is reflected in its interaction with nonnative proteins. To obtain insight into the chaperone activity of Hsp17 towards the *C. elegans* proteome, we incubated lysates from nonsynchronized *C. elegans* cultures at 37 °C and determined the effects of Hsp17 on the soluble and insoluble protein fractions. SDS-PAGE analysis showed that incubation at 37 °C resulted in the accumulation of insoluble lysate protein (Fig. 3*A*). Hsp17 was found in both, the soluble and insoluble fraction at all conditions studied, where 70% of Hsp17 remained in the soluble fraction. Interestingly, increasing amounts of Hsp17 did not suppress the formation of the insoluble fraction but rather led to an increase (Fig. 3, *A* and *B*). This indicates that Hsp17 is able to form both, soluble and insoluble complexes with substrate proteins. In contrast, Hsp16.2 mostly remained in the soluble fraction (Fig. S6*A*) and did not show any chaperone activity towards the endogenous proteins (Fig. S6, *A* and *B*).

Changes in the insoluble and soluble fractions were investigated in detail by MS analysis (Fig. S7). Four hundred fifty proteins proteins are overrepresented in the insoluble fraction when Hsp17 is present. In contrast, only 28 proteins are underrepresented, which correlates well with the *in vitro* assay results. The addition of Hsp16.2 resulted in smaller changes (84 proteins overrepresented/48 underrepresented), which is consistent with minimal changes in the soluble/insoluble fractions as analyzed by the SDS-PAGE. The presence of Sip1 led to 447 overrepresented and 52 underrepresented proteins (Fig. S7*A*). Interestingly, Hsp17 and Sip1 share an overlap of 279 proteins (approximately ∼60%) that are overrepresented in their insoluble fraction (Fig. S7*B*), while Sip1 and Hsp16.2 show only a small overlap. Moreover, the similarity between Hsp17 and Sip1 appeared clearly in the Pearson correlations of 0.813 between the insoluble fractions. In contrast, Hsp17 and Hsp16.2 (insoluble) showed a Pearson correlation of 0.452. For Sip1 and Hsp16.2 (insoluble), the Pearson correlation is even lower, 0.209. Comparison of the soluble fraction only revealed weak Pearson correlations (Fig. S7*C*).

In chaperone assays using a model substrate protein, coaggregation was observed with malate dehydrogenase (MDH) as evidenced by an increase in light scattering in the presence of Hsp17 (Fig. 3*E*) and consistent with previous results (39, 44). However, for citrate synthase (CS), another well-studied model substrate protein, Hsp17 prevents temperature-induced aggregation in a concentration-dependent manner (Fig. 3*C*). However, when we performed the chaperone assays with Hsp16.2, we observed a suppression of aggregation for both CS and MDH (Fig. 3, *D* and *F*). Thus, the observed substrate-dependent presence of both the holdase and aggregase activity for Hsp17 is a specific functional feature.

**Figure 4:**
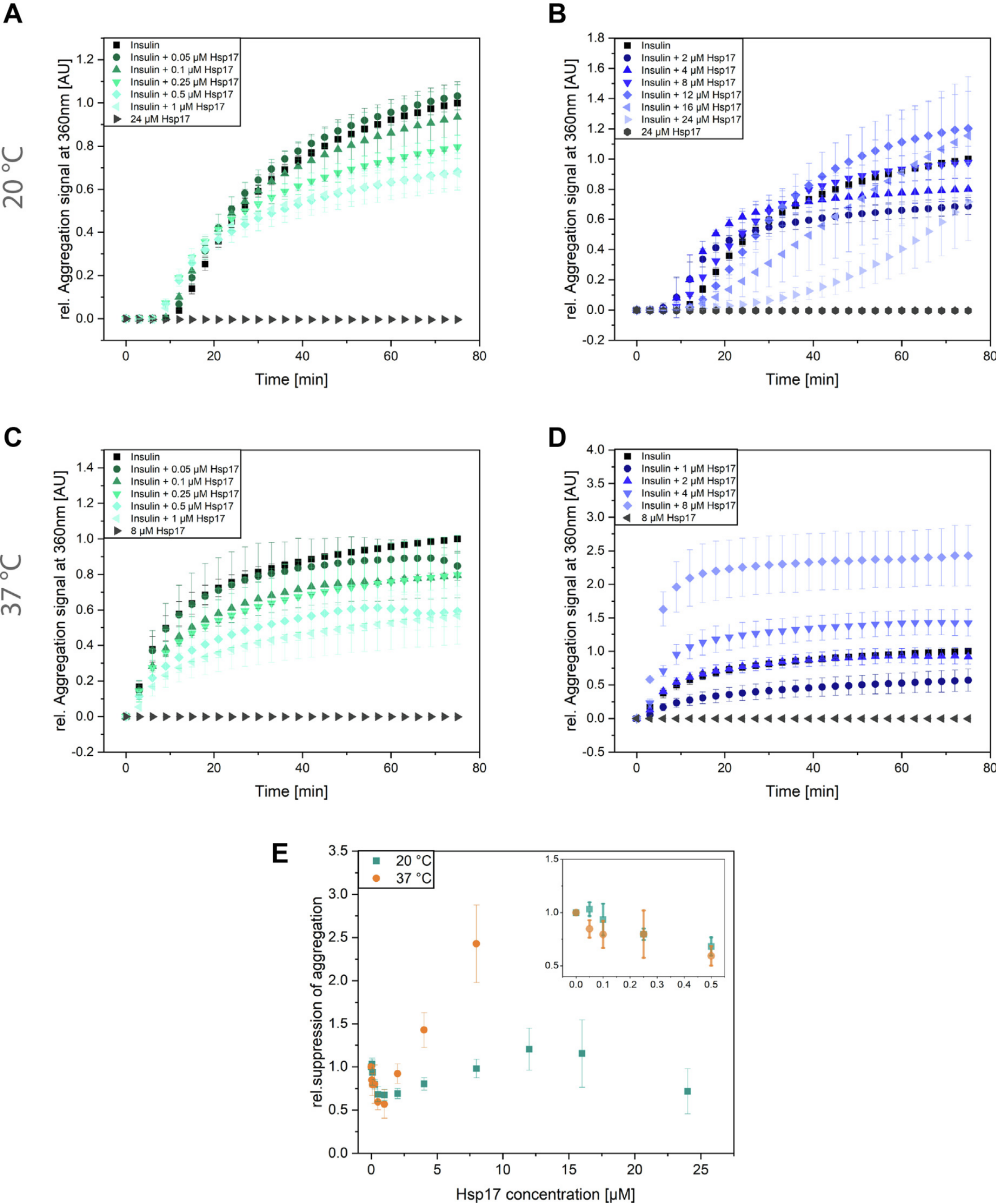
**Temperature- and concentration-dependent chaperone activity of Hsp17.** Chemical-induced aggregation of 40 μM insulin with various Hsp17 concentration 0.1 μM to 24 μM (*A*–*B*) at 20 °C (n = 3; error bars show SD) as well as at 37 °C with a range from 0.1 μM to 8 μM (*C*–*D*) Hsp17 (n = 3; error bars show SD). Under these conditions, Hsp17 showed a mild holdase activity when added in low μM concentrations, while its aggregase activity outweighs at concentrations higher than 2 μM. *E*, quantification of chaperone assay with insulin. All conditions were normalized to the aggregation signal of insulin after 75 min at the respective temperature (n = 3; error bars show SD).

To gain further insight into the potential substrate dependence of Hsp17’s chaperone mechanism, we carried out aggregation assays with the model substrate insulin (49, 50, 51). As unfolding of insulin is induced by the reduction of its disulfide bonds (52, 53), this assay can be performed at different temperatures (Fig. 4). This allowed us to ask the question whether elevated temperatures activate the chaperone function of Hsp17. When present at low concentrations (up to ∼2 μM at 20 °C and 8 μM at 37 °C), Hsp17 suppressed insulin aggregation in a concentration-dependent manner (Fig. 4, *A*, *C* and E). Notably, when Hsp17 is present at higher concentrations (*e.g.* ∼4 μM at 20 °C and ∼12 μM at 37 °C), the turbidity signal increased, indicating the coaggregation with insulin. Thus, at low Hsp17 concentrations, its holdase activity is dominating, while at higher concentrations, its aggregase activity is prevailing. From these experiments, we further conclude that elevated temperatures are not needed to activate Hsp17, which is consistent with the proteome interaction profiles obtained.

Interestingly, addition of higher amounts of Hsp17 slowed down insulin aggregation in a concentration-dependent manner, but during the time course of the reaction, aggregation started to increase both at 20 °C and 37 °C (Fig. 4, *B* and *D*). Thus, the two different chaperone modes, formation of soluble complexes or coaggregation, depend not only on specific substrate proteins but they also rely on the substrate to chaperone ratios for given substrate proteins. This implies that different complexes between a given substrate protein and Hsp17 can be formed. This feature is specific for Hsp17 and is not a general feature of *C. elegans* sHsps as revealed by the aggregation assays with Hsp16.2, in which the insulin aggregation was suppressed at any concentration and temperature (Fig. S6, *C* and *D*).

**Figure 5:**
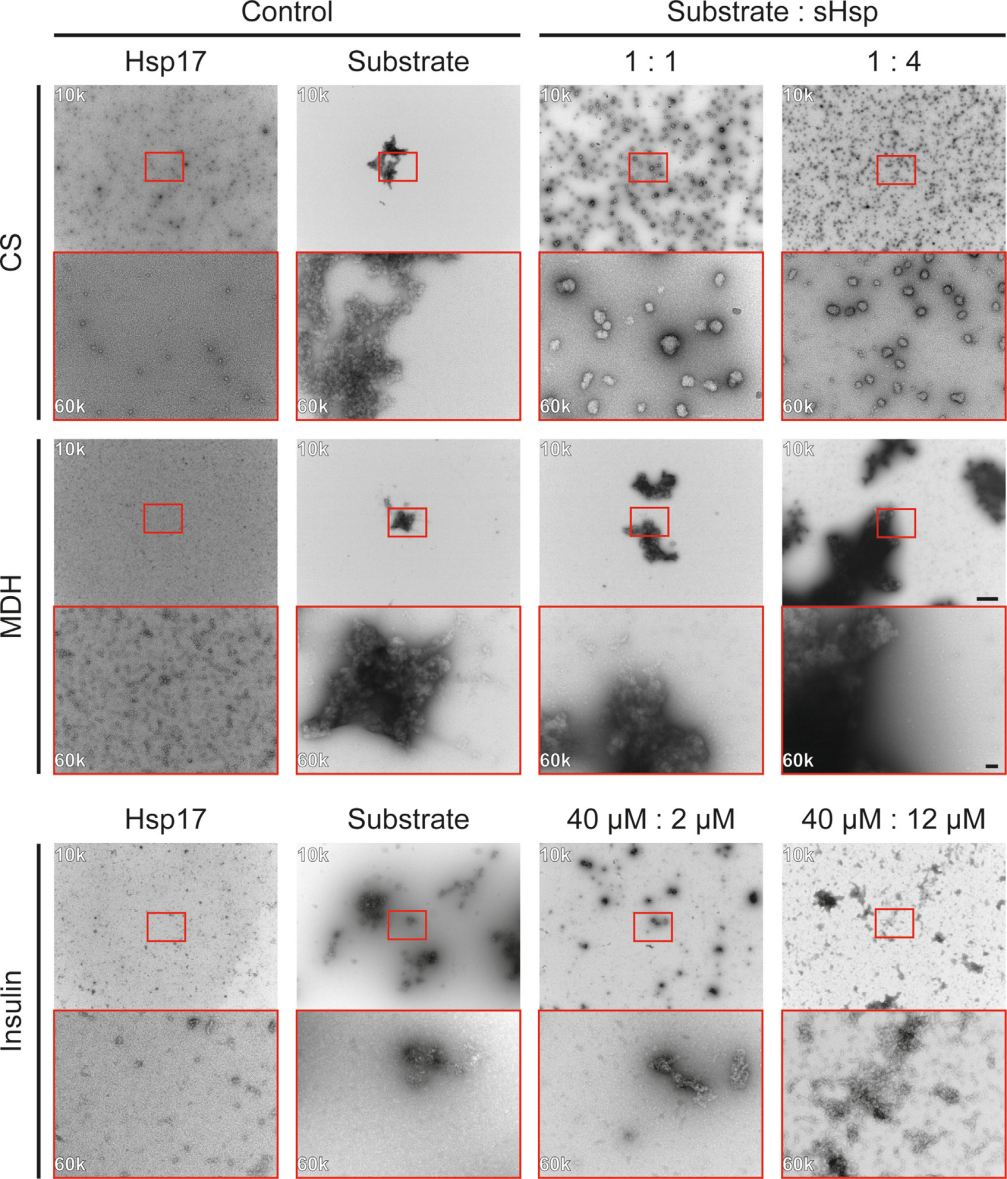
**Negative-stain EM of aggregation assay samples with different substrates.** Micrographs of Hsp17 with CS (*top row*), MDH (*second row*), and insulin (*bottom row*) as substrates in chaperone activity assays. The first two columns show Hsp17-only and substrate-only controls. Columns three and four display the endpoints of the activity assays with increased Hsp17 ratios. The same Hsp17 concentrations as in the corresponding chaperone activity assays were used. Samples were taken either after 30 min of incubation (CS and MDH) or 75 min (insulin). Additional time points and concentrations have been analyzed for the chaperone assay with insulin, see Fig. S8. Micrographs were acquired at 10k (*upper images*) and 60k (*lower images*) magnification. The areas in *red boxes* show the same part of the sample. Scale bars represent 500 nm and 50 nm, respectively. For each state, two independently prepared grids and at least 5 gridsquares per grid were analyzed. The chosen images represent the typical view during the analysis. MDH, malate dehydrogenase; CS, citrate synthase.

The structural analysis of Hsp17 by negative stain EM under assay conditions revealed that the wt oligomers have a size of roughly 12.6 nm at 20 °C and 37 °C in the absence of substrate proteins (Figs. S3*B* and 5, first column). In samples containing only the respective substrate protein at 37 °C, large amorphous aggregates were observed (Fig. 5, second column).

In the presence of Hsp17 and CS (1:1 molar ratio), globular complexes of Hsp17 and CS with a size of approximately 30 to 80 nm were formed. At a CS:Hsp17 ratio of 1:4, the globular appearance persists, while the size of the complexes is reduced to approximately 10 to 50 nm and their quantity is increased (Fig. 5, top row). A different behavior was observed in the presence of MDH (Fig. 5, second row). Here, no defined MDH–Hsp17 complexes were visible. The structures observed were comparable to that of the amorphous aggregates detected for MDH alone. Nevertheless, the amount of Hsp17 oligomers declined, indicating its incorporation into the aggregates. Increasing amounts of Hsp17 led to an increase in aggregate sizes. This is consistent with the increase in turbidity observed in the MDH chaperone assays in the presence of Hsp17. Aggregation assays with insulin at 20 °C showed the formation of smaller, more compact aggregates at low concentrations of Hsp17. At high Hsp17 concentrations, large unstructured aggregates were present (Fig. 5, bottom row). Both at 20 °C and 37 °C, samples with low Hsp17 concentrations showed reduced insulin aggregates than the presence of higher Hsp17 concentrations. Additionally, the increase of aggregate size during the experiment is lower at higher Hsp17 concentrations. (Figs. S8 and S9).

Thus, both the formation of soluble substrate complexes and coaggregation is part of the chaperone mechanism of Hsp17.

### The cryo-EM structure of Hsp17 reveals a bipartite organization of the NTRs

**Figure 6:**
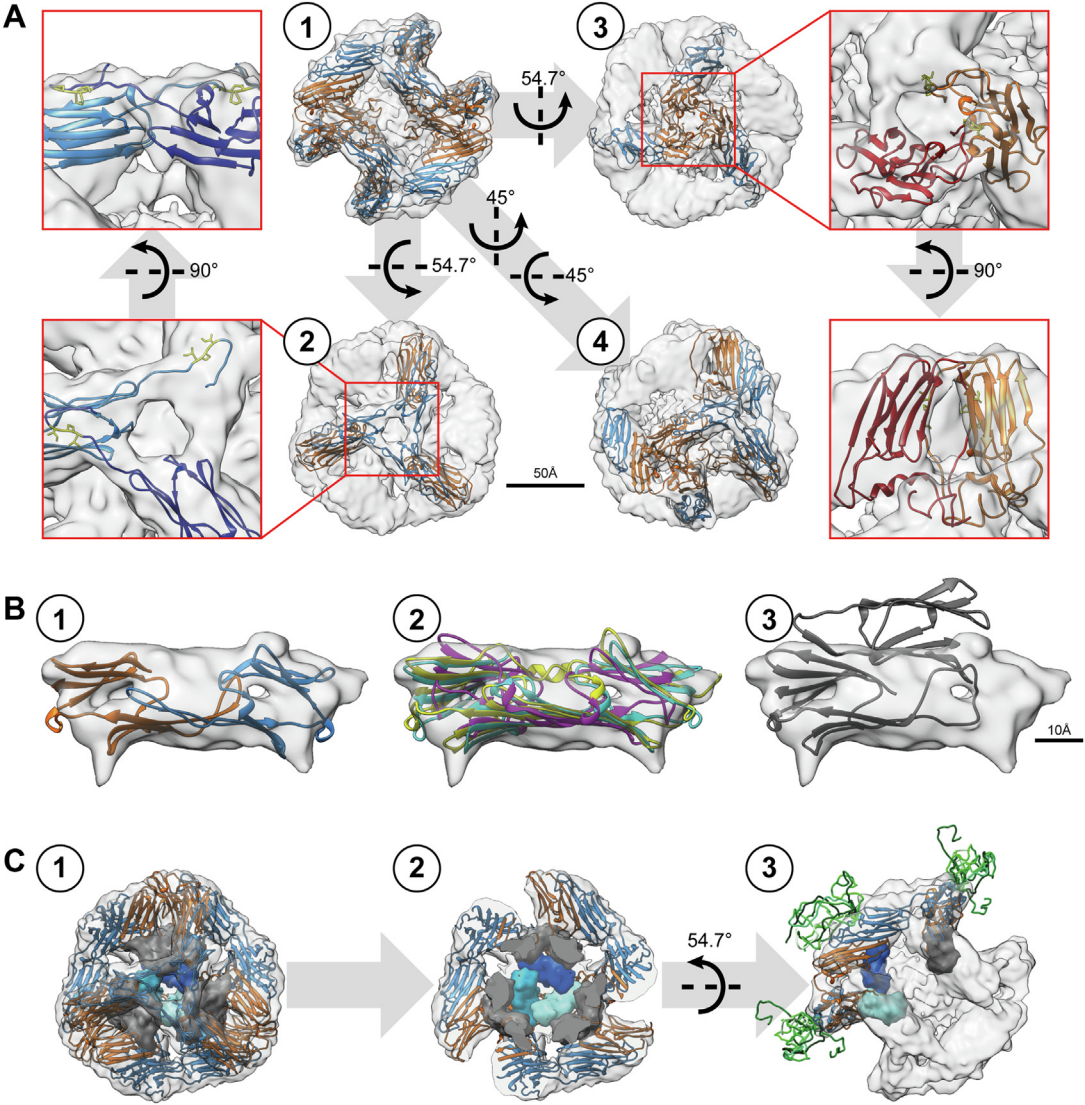
**Cryo-EM structure of Hsp17.***A*, reconstruction of Hsp17 showing the tetrameric oligomer structure as transparent map with fitted pseudo-atomic model. Looking at the face of the oligomer (*A1*), one can rotate the model by 54.7° in either the x or y direction to visualize the region where 3 monomers interact (*A2* and *A3*). The *red areas* are shown in the insets with only two monomers, where one is colored in a darker shade for better differentiation of the two interaction modes. The I-x-I motif is depicted in *yellow*. Two consecutive rotations by 45° in x and y direction lead to a view (*A4*) where the dimeric building block of the oligomer with its contacting points to four different dimers can be discerned. *B*, detailed view of the Hsp17 dimeric building block. The density map has been thresholded to a value that allows for good visibility of the beta-sheets. *B1*, structure of Hsp17 ACD fitted into the density with the two monomers colored in *orange* and *blue*. *B2*, fitting of structures of Sip1 (4ydz; *yellow*), αA-crystallin (3l1f; *cyan*), and αB-crystallin (3l1g; *magenta*) into the density map. *B3*, fitting of the wheat Hsp16.9 forming the nonmetazoan fold with respect to the visible beta-sheet density. *C1*, oligomer reconstruction with pseudo-atomic structure fitted into the density. Inward-facing NTRs are shown as opaque densities, where the three *blue* densities illustrate one possible interaction site at the oligomer corner. The remaining nine NTRs are shown in *gray* to represent their space requirement. *C2*, oligomer from (*C1*) clipped in half to offer a better view on the NTR interactions. *C3*, view along the 2-fold symmetry axis with 3 fitted dimers in *orange*/*blue* and the three corresponding inward-facing NTRs shown as opaque densities. Additionally, surface NTRs are shown as a superimposition of 10 structures in various *green* hues to represent their space requirement. ACD, α-crystallin domain; NTR, N-terminal region.

To address the basis for the special chaperone mechanism of Hsp17, we solved the structure of the Hsp17 wt 24-mer by cryo-EM (Fig. 6 and Table S1). The oligomer has a cubic shape with the symmetry of a regular tetrahedron (Fig. 6*A*). The edges of the cube are represented by ACD dimers resulting in a 24-mer structure. In this symmetric structure, we were able to fit the rigid ACD precisely into the reconstruction. Furthermore, we could localize the NTR and the CTR in the oligomer. In line with findings on other sHsps (49), the overall resolution of the EM reconstitution is 6.49 Å with the different parts of Hsp17 differing in resolution. While the rigid ACD shows a resolution below 5 Å, the CTR could only be resolved to 7 Å due to its inherent flexibility. The NTR was partly visible with a resolution of about 9 Å (Fig. S10*A*). Contrary to sHsps like αA-crystallin (54) or Sip1 (24), we could not detect different oligomeric species for Hsp17.

An important point for the assembly of sHsp oligomers is the mode of association of the ACD dimer. There are two different types: the nonmetazoan type is characterized by the interaction of the β2 strand of one monomer with the β6 strand of its partner monomer (β6-swapped dimer) leading to the formation of four β-sheets (55). The metazoan fold consists of an antiparallel interaction of the β6-7 strand from both monomers (β7-interface dimer) resulting in one large and two smaller β-sheets. The resolution achieved allowed us to identify the location of the β-sheets in the Hsp17 dimer and to unambiguously position the ACD into the reconstruction. Fitting the structure of a crystallized sHsp ACD with a nonmetazoan fold (*e.g.* wheat Hsp16; 1gme) leads to a poor cross-correlation of 0.69, while structures of ACDs with a metazoan fold show significantly higher cross-correlations: 0.89 (*C. elegans* Sip1; 4ydz), 0.89 (human αA-crystallin; 3l1f), 0.88 (human αB-crystallin; 3l1g), and 0.86 (rat Hsp20; 2wj5) (Fig. 6*B*). Thus, in contrast to previous results (44), our reconstruction shows that Hsp17 adopts the metazoan ACD dimer interface. This is further underpinned by the superposition of our reconstruction with the AlphaFold prediction (Q20660; Fig. S10*B*) (56). Additionally, we identified crosslinks (Fig. S9) between Lys91 and Lys84, Lys102, Lys127 and Lys130, as well as between Lys88 and Lys102 that confirm the metazoan ACD dimer interface (Fig. S11, *A* and *B*).

Due to their orientations in the oligomer, we were able to distinguish clearly between a surface-facing monomer (Fig. 6*A* blue) and an inward-facing monomer in the ACD dimers (Fig. 6*A* orange). The NTRs of the surface-facing monomers are oriented to the outside of the oligomer and are partly unresolved, whereas the NTRs of the inward-facing monomers protrude towards the inside of the oligomer and interact with each other. The CTRs connect the monomers at the corners of the cube, where three edges meet in two different modes depending on the type of monomer (surface- or inward-facing) according to the tetrahedral symmetry (Fig. 6*A* insets).

Due to the high conformational heterogeneity and flexibility typical for sHsps (16), further subclassification and focused classifications did not lead to an improvement of the structure. Instead, we performed an asymmetric classification into 50 classes to analyze conformational differences of the individual monomers in the oligomer structure. We were able to discern all possible variations of the CTR-mediated connections at the corners (Fig. S12). Both the surface monomers as well as the inward-facing monomers are involved in oligomer formation. Due to the cubic shape of the oligomer, either three surface monomers or three inward-facing monomers interact with each other at the oligomer corner (Fig. S12*A*) with one, two, or three of the involved CTRs creating the connection (Fig. S12*B*). Through classification of the asymmetric reconstructions, we could quantify the results for the formation of a one-, two-, or three-fold connection at the oligomer corner. Most of the oligomer corners (76.5%) form a three-fold connection, followed by the twofold connection with 21.7% and the onefold connection with 1.8% of the oligomer corners (Fig. S12*B*).

Our analysis revealed that two different binding modes of the CTR seen in the density map also exhibit two different types of connections between monomers in the pseudo-atomic structure: either surface-exposed or in the interior (Fig. 6*A*). In both types, the link between the monomers is established by the binding of the I-x-I motif of the CTR into the pocket formed by the β4 and β8 strands of the ACD of a neighboring monomer. Results for crosslinks of Lys residues in the CTR with residues from the connecting monomers support the two types of connections. The crosslinks between Lys91 and Lys137 as well as Lys91 and Lys146 are found in the connection between inward-facing monomers (Fig. S11*C*), whereas the crosslinks between Lys91 and Lys130, Lys102 and Lys146, Lys127 and Lys146, as well as Lys130 and Lys146 are characteristic for the connection between surface monomers (Fig. S11*E*). Since Hsp17ΔNTR forms only smaller oligomers, this CTR-mediated connection alone is not sufficient for the formation of large oligomers. The NTRs must therefore play an important role.

We were able to generate a pseudo-atomic model of the Hsp17 24-mer consisting of residues 25 to 148 for the surface monomer and residues 1 to 148 for the inward-facing monomer. The first 24 residues of the surface monomer did not result in a distinctive density and therefore were excluded from the pseudo-atomic model. Based on a homology model with Sip1 as a template, we flexibly fitted the structure into our refined density using molecular dynamics simulations and refined the structure iteratively.

Since the NTR of the surface monomers could not be resolved completely in the reconstruction, we assume high flexibility in this part of the molecule. This notion is further supported by results from crosslinking experiments where Met1 displays crosslinks to lysine residues all over the surface of the oligomer (Fig. S11, *A* and *F*). In the interior of the sphere, we identified a broad connection between the density occupied by the ACD and a central mass, which represents the twelve inward-facing NTRs (Fig. 6*C*). This density as well as the crosslink between Met1 and Lys146, which reflects connections between inward-facing monomers and the NTRs (Fig. S11*C*) suggests that the inward-facing NTRs come into close contact. These interactions are flexible as the local resolution of the central mass inside the symmetric reconstruction is low (Fig. S10*A*).

## Discussion

In the last decades, numerous studies using *C. elegans* as a model organism have unveiled exciting insights in the proteostasis network in disease (57, 58) and aging (21, 59, 60), with a special emphasis on the impact of molecular chaperones (61, 62). In this context, the large number of different sHsps in *C. elegans* begs the question what the specific contribution of each of them to the proteostasis network is.

Hsp17 is of special interest as it is not closely related to one of the other *C. elegans* sHsps. Its importance for *C. elegans* is demonstrated by the finding that Hsp17 is expressed in an increasing number of tissues during development. Further, Hsp17 is present in many tissues in adult nematodes under physiological conditions (39). Under stress, the Hsp17 levels are only slightly increased (40). These traits are unique among the *C. elegans* sHsps studied so far. Sip1 shows the highest expression in embryos (24). In later developmental stages, Sip1 expression is strongly diminished except for the gonadal arms (63). Hsp16s are expressed in multiple tissues upon heat stress, such as Hsp16.2 in the intestine and in the pharynx (36, 37) and Hsp16.48 in the intestine, body muscle, hypodermis, and pharynx (38). Thus, it seems that the expression patterns of the different sHsps in *C. elegans* show some overlap in the tissue distribution on the one hand, and on the other hand, also unique tissue expression patterns are observed that might explain the expansion of the sHsp family during evolution (24, 64). The Hsp17 expression pattern strongly suggests that in contrast to the Hsp16s, it might be required mainly under physiological conditions. This idea is supported by its mode of action and its substrate spectra.

Co-IPs from worm lysates revealed that Hsp17 interacts with hundreds of proteins as expected for the promiscuous binding characteristics of sHsps. The comparison with two other *C. elegans* sHsps, Hsp16.2 and Sip1, revealed a core set of common interactors and a large number of individual binders suggesting that in addition to tissue specificity, there is also substrate specificity. Of interest, all three sHsps show a shift concerning their interaction partners from 20 °C to 37 °C. Of note, although our results indicate that Hsp16.2 is also active at physiological temperatures, it is mainly expressed under stress in *C. elegans*.

The sHsps investigated seem to favor substrate proteins with a slightly higher median molecular weight than that representative for the entire *C. elegans* proteome consistent with the idea that larger proteins rely more on chaperones to maintain their structure than small ones (65). Interestingly, Hsp17 does not protect the cellular proteomes from aggregation, rather Hsp17 seems to coaggregate as the amount of insoluble protein increases with Hsp17 concentration. In contrast, Hsp16.2 left the overall protein amount in the insoluble fraction unchanged. A deeper analysis of the insoluble fractions via MS uncovered that Hsp17 and Sip1 share 60% of the overrepresented proteins. Hsp16.2 seems to be more distinct as less proteins are overrepresented. Besides that, there is a 58% overlap with the insoluble fraction of Hsp17 but only an 11% overlap with Sip1. In summary, the proteome data suggest that regarding changes in the insoluble fraction, Hsp17 and Sip1 showed higher similarities than Hsp16.2 and Sip1 as it is the case for the Co-IP samples. This does not mean that the insoluble proteins are irreversibly lost. Rather this could enable an alternative way to stabilize the proteome under stress conditions, for example, via sequestration. It has been shown previously for yeast and mammalian cells that also aggregates containing sHsp–substrate complexes can be efficiently resolved by Hsp70 systems (20, 22, 23, 66, 67). However, since Hsp17 is not exclusively found in the insoluble fraction, the formation of soluble complexes also seems to contribute to proteostasis in the *C. elegans* cytosol. Thus, substrate proteins found in soluble or insoluble complexes may be amenable to refolding.

*In vitro*, most sHsps studied form defined substrate complexes at (sub)stoichiometric ratios of substrate protein *versus* sHsp and insoluble aggregates together with the substrate protein when the substrate is present in excess (19, 66). However, for Hsp17, either coaggregation or suppression of aggregation was observed for different model substrates. Thus, the nature of the substrate protein seemed to play the decisive role concerning the outcome of the chaperone reaction (39). However, in the case of insulin, both the formation of soluble complexes and the formation of aggregates were detected for the same protein—depending on the ratio and the temperature. Thus, the specific conditions of the chaperone reaction (*e.g.*, ratio of substrate:sHsp, temperature) are important in determining the outcome. It is tempting to speculate that one of the key traits of Hsp17, the presence of mono-disperse, permanently active species (24mers) is responsible for the difference in chaperone function compared to other sHsps. In the case of sHsps exhibiting ensembles of oligomers differing in size, with smaller oligomers being more active, the coaggregation may be prevented more efficiently.

These experiments also revealed that in contrast to some other sHsps (24, 68), Hsp17 does not need to be activated to unleash its chaperone activity. However, our analyses do not address the question whether further mechanisms for modulating the activity of Hsp17, such as posttranslational modifications, exist. Furthermore, Hsp17 does not form an ensemble of oligomeric states as observed for other sHsps, rather the 24mer present under all conditions tested is the active species (39, 44). We could not detect the formation of supermolecular Hsp17 assemblies as previously reported (44). It may well be that the His-tag present in the construct used in the previous study affects the assembly.

One of the main driving forces for oligomerization of Hsp17 is the NTR, as the variant lacking the NTR does not form large oligomers; only smaller species consisting of 2 to 4 monomers were observed. The difference in thermal stability between Hsp17 and Hsp17ΔNTR which is in line with findings for other sHsps (69) as well as the higher HD exchange of Hsp17ΔNTR compared to the full-length protein supports the assumption that NTR interactions are important for stabilizing the 24mer.

How the structural features of Hsp17 are connected to its distinct chaperone characteristics was revealed by the cryo-EM structure determined in this study. The oligomer has a diameter of roughly 12.6 nm and only the tetrahedral 24-mer was observed. Polyhedral arrangement have been previously observed among sHsps (70, 71). It should be noted that the size distribution shows a peak between 12 and 14 nm in diameter. From a geometric point of view, this matches the sizes of the face diagonal and the space diagonal of a cube.

This is different from the well-characterized *C. elegans* Sip1, which forms different-sized oligomers with various dihedral symmetries (6- to 8-fold) (24). In both sHsps, two monomers interact via the β7-interface dimer characteristic for metazoan sHsps and not via the β6-swapped dimer as previously suggested for Hsp17 (44). The ACDs are connected via the C-terminal I-X-I motifs which bind to the pocket formed by the β4 and β8 strands of the neighboring ACD. The CTRs on both ends of the dimer show different conformations, resulting in different connections (24). In Hsp17, this feature discriminates the two different oligomer corners (between inward-facing monomers or surface monomers).

NTRs are often not resolved in sHsps structures due to their flexible nature (10, 72, 73, 74). For several sHsps including Sip1, the NTRs have been described to be buried in the inside of the structure (24, 49, 72, 75). However, in the structure of Hsp17, there is a bipartite distribution with 12 NTRs located on the surface of the oligomer and 12 NTRs pointing towards the center of the oligomer. The surface NTRs are flexible and could not be resolved, while the inside NTRs are represented by a defined density. Consistent with their flexibility, the surface NTRs could be crosslinked to various lysine residues on the surface of the oligomer. The outward-facing NTRs are positioned ideally to engage in substrate–protein interactions, while the ones located in the interior interact with each other and stabilize the oligomer. Thus, the same segment fulfills two different functions depending on the structural context in the Hsp17 oligomer.

The organization of the Hsp17 NTRs and the unique presence of a 24-mer structure are different from previously described sHsp oligomer structures (24, 75, 76). The common view has been that sHsps form ensembles of large oligomers governed by NTR interactions. As a result of this, in larger oligomers, NTRs are engaged in homotypic interactions regulating the oligomeric state, while the NTRs in smaller oligomers are free to play a role in substrate interaction (77). Accordingly, the large oligomers are less active as molecular chaperones than the smaller species (78). Hsp17 does not fit into this scheme: it is present only in one oligomeric form, the 24-mer, and it does not seem to require dissociation for activation of the chaperone function. Hsp17’s unique trait is based on the specific arrangement of the NTRs which functionally separates them into two groups: those involved in formation and stabilization of the 24-mer in the inside of the cube and those accessible for substrate on its outside. This creates a permanently chaperone-active sHsp consistent with the view that it performs household functions under physiological conditions. We did not obtain evidence that dissociation occurs at increased temperature but in principle activation of Hsp17 through dissociation cannot be ruled out, for example, induced by posttranslational modifications.

It is reasonable to assume that the specific structural and chaperone signature of Hsp17, which results in a distinct protection of the cellular proteome, has also been employed in other sHsp family members outside the phylum of nematodes.

## Experimental procedures

### Bioinformatics

All sequences were obtained from uniport.org (79). Alignment, phylogenetic tree, and percent identity matrix were calculated with Clustal O with default settings (80, 81). Visualization of the alignment and the phylogenetic tree was done with Jalview (82) or iTOL (83), respectively. Pfam family Pf00011 (84) was used to identify sHsps of *C. elegans* in Uniprot. The position of the ACD was identified via sequence alignment with Sip1, and JPred4 was used to identify secondary structure elements (85). The Sequence logo was generated with WebLogo online tool (86).

### Cloning and purification

Hsp17 and Hsp16.2 was cloned from cDNA into pET21a (Novagen) via NdeI and XhoI. For Hsp17ΔNTR, the endogenous sequence starts at aa position 45 with an added Met in front of it. To enabling tag-free expression, a stop codon is added in front of the XhoI cleavage site. Recombinant protein expression was performed in *Escherichia coli* BL21 (DE3) in LB-Lennox medium (Serva) at 37 °C until an OD_600_ around 0.7 was reached. Expression was induced with 1 mM IPTG and the cultures were shifted to 25 °C overnight for Hsp17 and Hsp17ΔNTR, whereas for Hsp16.2, cultures were kept at 37 °C. Cells, expressing Hsp17 and Hsp17ΔNTR, were harvested the next day and washed with low salt buffer (50 mM Tris, 5 mM EDTA, 20 mM NaCl, pH 8.0). For cell disruption, pellets were resuspended in low salt buffer with protease inhibitor mix G (Serva), and a Basic Z (Constant Systems) cell disruptor was used at a pressure of 1.8 kbar. Subsequently, DNaseI were added, and the lysate was centrifuged for 45 min at 39, 000*g*. The soluble fraction was directly loaded on a Sepharose Q FF (GE Healthcare). Hsp17 was eluted with a salt gradient from 20 mM to 1 M NaCl over 5 column volumes. Hsp17-containing fractions were loaded on a Superdex 200 pg (GE Healthcare). For elution, a 150 mM salt buffer was used (50 mM Tris, 5 mM EDTA, 150 mM NaCl, pH 8.0). The expression of Hsp17ΔNTR and the purification was identical to the above-described procedure with the exception that the second column was replaced by a Superdex 75 pg (GE Healthcare). Sip1 purification was performed as previously described by (24) with some slight modification. As protease inhibitor, we used the inhibitor mix G (Serva). Additionally, the third purification step was performed on an HiTrap Q instead of a Resource Q. Lastly, as storage buffer, we used 50 mM Tris, 5 mM EDTA, 150 mM, and 1 mM DTT (pH 8.5). Recombinant Hsp16.2 was purified from inclusion bodies. After cells were harvested by centrifugation, they were resuspended in ice cold IB buffer (50 mM Tris (pH 7.4), 10 mM EDTA, 50 mM NaCl, protease inhibitor mix G, pH 7.4). Cell disruption was also performed with the cell disruption system Basic Z. One percentage of Triton X-100 was added to the lysate and stirred for 1 h at 4 °C. Inclusion bodies were harvested by centrifugation at 48, 250*g* at 8 °C and washed with IB buffer at least three times. Next, inclusion bodies were resuspended in IB dissolving buffer (50 mM Tris; pH 7.4, 5 mM EDTA, 4 M Urea, 10 mM β-Mercaptoethanol) and dissolved by stirring for 2 h at room temperature. Afterward, insoluble components were removed via centrifugation at 48, 250 °C at 25 °C. The supernatant was applied to a Sepharose Q FF column equilibrated in 50 mM Tris (pH 7.4), 5 mM EDTA, 50 mM NaCl, 5 mM DTT, 4 M urea and eluted using a salt gradient up to 1 M NaCl. The second purification step was performed with a Sepharose SP by using the same buffer as for the first purification step. Final polishing was done with a Superdex 75 pg column equilibrated in 50 mM Tris, pH 7.4, 5 mM EDTA, 150 mM NaCl, 5 mM DTT, 4 M GdmCl. Refolding was performed in PBS on a Superdex 200 pg, and fractions containing Hsp16.2 were collected and dialyzed overnight against PBS which was also used as the storage buffer. Purity of all purified proteins was checked by SDS-PAGE and the concentrations were determined by absorption using coefficients, which were calculated with the ProtParam tool (87).

### CD spectroscopy

To investigate the secondary structure of the proteins, CD measurements were performed, using a Chirascan plus spectropolarimeter (Applied Photophysics). Quartz glass cuvettes (SUPRASIL grade, Hellma Analytics) with an optical pathway of 0.2 or 0.5 mm were used. Far UV spectra were recorded at 20 °C. Measurements were done with a protein concentration of 0.25 to 0.3 mg/ml. Ten repeats were recorded, and the average was calculated. To analyze their thermal stabilities, protein solutions were heated with a rate of 1 °C/min.

### SEC measurements

SEC analysis was done with an HPLC system (Shimadzu) and a Superdex 200 Increase 10/300 Gl column (GE Healthcare). All measurements were performed in PBS and the system was operated with a flow rate of 0.5 ml/min at room temperature. The sample volume was 50 μl with a protein concentration of 50 μM.

### SDS-PAGE and native PAGE

For the SDS-PAGE separation of *C. elegans* lysates, TG Prime 4 to 20% gels (Serva) were used. Samples were incubated for 8 min at 95 °C in Laemmli buffer before loading on the gel. The running buffer contained 25 mM Tris, 200 mM Glycin, 0.1% SDS, pH 8.8. For native PAGE, samples were incubated for 30 min at different temperatures. Immediately after the incubation step, sample buffer for clear native gels (Serva) was added, and samples were loaded on 4 to 16% native gels (Serva) and separated using native anode and cathode buffers from Serva.

### HDX-MS measurements

To determine HDX-MS, a HDX-MS system from Waters, consisting of a Synapt G2-Si MS, ACQUITY M-Class UPLC, HDX Manager, and a LEAP HDX-2 automation platform, was used as described previously (54, 88). Measurements were performed with an initial protein concentration of 30 μM which was diluted in a 1:20 ratio into PBS with D_2_O. Samples were incubated for 10, 60, 600, 1800, and 7200 s. As 0 s, the experiment was performed without Deuterium. HD exchange was stopped with a quenching solution (200 mM Na_2_HPO_4_, 200 mM KH_2_PO_4_, 4 M GdmCl, pH 2.3) in a 1:1 ratio at 1 °C. The sample was directly transferred on a digestive Pepsin Column (Waters Enzymate BEH Pepsin Column 2.1 mm × 30 mm). The pepsin digestion took place at 0 °C. Peptides separated by chromatography using an ACQUITY UPLC BEH C18 1.7 μM VanGuard Pre-Column 2. 1 × 5 mm, followed by an analytical column (ACQUITY UPLC BEH C18 1.7 μM 1.0 × 100 mm) before peptides were measured by MS. Analysis of the HDX data was done with DynamX (Version 3.0).

### Negative stain EM

Five microliters of the protein solution were applied to glow-discharged copper grids with continuous carbon film (20 nm thickness, prepared in the lab) after heat shock. Excess solution was blotted from the grid after 60 s adsorption time, and 5 μl stain solution (2% wt uranyl acetate) were applied immediately. After 30 s, the remaining stain solution was blotted away and the grid was allowed to dry. The grids were transferred to a JEOL JEM-1400Plus microscope equipped with a JEOL Ruby CCD camera and image acquisition was performed at 120 kV acceleration voltage at nominal magnifications of 10k and 60k.

### Cryo-EM

Four microliters of the protein solution were applied to hydrophilized Quantifoil R2/1 holey carbon grids and plunge frozen using a FEI Vitrobot (Mark IV) with liquid ethane at −183 °C. The protein concentration was 2.5 mg/ml and blotting time was adjusted between 5 and 10 s to optimize ice thickness for automated image acquisition. The grids were transferred to an FEI Titan Krios microscope equipped with a GATAN K3 camera operating in CDS mode. Two thousand five hundred sixty-eight micrographs were automatically acquired at 300 kV acceleration voltage and a nominal pixel size of 1.09 Å in a defocus range of −0.5 μm to −2.5 μm. The raw image stacks contained 30 frames with a total dose of 55 e/Å^2^.

### Image processing

Image frames were motion corrected and dose weighted with MotionCor2 (89), further analyzed using Gctf (v 1.06) (90) and an initial particle set was generated using template-free gautomatch particle picking inside RELION (91). After migrating the dataset to cryoSPARC2 (92), 2D classes were used in a subsequent supervised picking as templates to improve picking results yielding 663,141 particles. Two additional rounds of 2D classification were used to remove falsely picked particles. The remaining 344,489 particles were utilized to classify into three initial models with C3 symmetry (*Ab initio* reconstruction). The best class (consisting of 187,116 particles) was further improved via homogeneous refinement with tetrahedral symmetry generating the symmetrical reconstruction presented in this study. Here, the FSC shows a sudden dip at around 12 Å, which is caused by the flexible and poorly resolved center of the oligomer where dynamic masking cannot be applied properly. To address the remaining heterogeneity in the dataset, we used symmetry expansion with tetrahedral symmetry and extensive 3D classification with local alignment in RELION to generate 50 classes. 3D structures were visualized with UCSF Chimera (93). Contour levels are calculated to represent the protein volume of all resolved amino acids.

### Structure modeling

A homology model of Hsp17 was generated using I-TASSER (94) with the closely related sHsp Sip1 (4ydz) as initial template. After rigid body fitting the model into the reconstruction using UCSF Chimera, we continued fitting the structure flexibly using molecular dynamics via NAMD2 (95). Therefore, multiple steps of MDFF (96) simulations in VMD (97) were calculated in vacuum at 300 K to 320 K with the CHARMM force field (98) and variable scaling of the grid forces (0.7–0.3). Finally, the structure was iteratively refined using the program phenix.real_space_refine (99) optimized for the use with cryo-EM data inside the PHENIX suite (100). Remaining Ramachandran- and sidechain-outliers were manually corrected using Coot (101).

### Chaperone activity assays

Chaperone assays, measuring thermal-induced aggregation, were performed with two different substrates: MDH (mitochondrial MDH from porcine heart, Roche) and CS (from porcine heart, Sigma-Aldrich). For CS, a 500 nM concentration in 40 mM Hepes (pH 7.4) was chosen and the assay temperature was set to 42 °C. Whereas for assays with MDH, the concentration was 1 μM in PBS at 45 °C. To measure the chaperone activity for Hsp17 in the presence of chemical inactivated substrate, insulin was used. A 40 μM insulin concentration was selected and its aggregation was induced by the addition of DTT (20 mM) after 5 min preincubation with the sHsp. Turbidity of the protein solution was measured at 360 nm with a Cary50 (Varian), and quartz glass cuvettes (SUPRASIL grade, Hellma Analytics) were used. Samples for subsequent analysis by negative stain EM were taken after 30 min incubation from chaperone assay with CS and MDH. From assays with insulin, samples were taken after 25, 50, or 75 min as indicated.

### Culture of *C. elegans*

Worms were cultivated either on NGM agar plates as described previously (102), or, to obtain a sufficient amount of *C. elegans* lysate, worms were cultured in S Basal Medium (103) or on NGM agar plates. N2 Bristol worms were used and the Hsp17^−/−^ strain (*tm5013*) was obtained from Dr S. Mitani/NBRP (104) and validated via PCR.

### Production of *C. elegans* lysates

Nonsynchronous lysates were harvested from liquid culture and washed with M9 buffer (6.0 g/l Na_2_HPO_4_, 3.0 g/l KH_2_PO_4_, 5.0 g/l NaCl, 1.0 g/l, and 1 mM MgSO_4_). To get rid of OP50 *E. coli*, worms were resuspended in a 30% sucrose solution and centrifuged at 3170*g* for 5 min. Floating worms were immediately aspirated and transferred to M9 buffer, spun down, and resuspended in 200 μl worm lysis buffer (15 mM Mops, 15 mM Mes, 150 mM NaCl, 2 mM EDTA, 1% (v/v) NP-40, 0.5% (w/v) sodium deoxycholate, 1 mM PMSF, 1% Serva Protease Inhibitor Mix M, pH 7.5). The worms were grinded with a glass mortar and transferred to an Eppendorf cup. Next, 200 μl glass beads (diameter 0.25–0.5 mm, Carl Roth) were added and the worms crushed with a mixer mill (Retsch GmbH) at a frequency of 30 s^−1^ for 1 min. This procedure was repeated three times, with a 1 min incubation step on ice in between. Glass beads were removed via centrifugation at low speed (510*g*) before lysates were cleared from debris by centrifugation at 16,900*g* for 20 min.

### Microinjection of *C. elegans*

The Fosmid pCC1FOS, containing the genomic DNA of Hsp17, was obtained from the *C. elegans* TransgeneOme Resource (CBGtg9050C1126D; Source BioScience). This vector contains around 25 kbp upstream and 15 kbp downstream of Hsp17’s chromosomal sequence. After the coding sequence of Hsp17, the stop codon was removed and a GFP sequence was inserted, leading to the expression of Hsp17::GFP. Microinjections were performed with the electronic microinjector FemtoFet express (Eppendorf). The injection mixture contained 0.5 ng/μl Fosmid DNA, 150 ng/μl pRF4 in 20 mM potassium phosphate, 3 mM potassium citrate, and 2% PEG 8000 (pH 7.5). This mixture was injected into the gonads of young N2 adults, the F1 roller were singled, and the following generation was analyzed.

### Microscopic analysis of *C. elegans*

To enable imaging, worms were immobilized with 150 mM sodium azide added to a modified M9 buffer (5.8 g/l Na_2_HPO_4_, 3.0 g/l KH_2_PO_4_, 0.5 g/l NaCl, 1.0 g/l) on a 2% agarose pad. Fluorescent images were recorded with an Axioscope 2 (Zeiss) equipped with a charge-coupled derive camera (Micromax) and a 63 × 1.4 NA oil lens. Images were recorded with MetaMorph (version 7.1). All images showing whole worms were recorded with a Leica M205FA using the software Leica Application Suite (version 3.2.0.9652).

### Lysate aggregation assay

Lysate aggregation assays were performed with *C. elegans* lysate from Hsp17^−/−^ worms in PBS buffer. The used lysate concentration was 0.8 mg/ml and different amounts of sHsps were added. Experiments were carried out in a total volume of 50 μl for analysis via SDS-PAGE. The sample volume was adjusted to 100 μl for later analysis via mass spectrometry, and the sHsps concentration was adjusted to 8 μM. Lysate-sHsps mixtures were incubated for 90 min at 37 °C. Following this, samples were centrifuged at 10,000*g* for 10 min. The supernatant was removed carefully and further used as the soluble fraction. To remove the rests of soluble protein, the pellets were washed three times with 200 μl PBS with centrifugation steps at 10,000*g* for 10 min. Pellets were finally solubilized in 1× Laemmli buffer and incubated for 8 min at 95 °C. The whole insoluble fraction and half of the soluble fraction was loaded.

### Co-IP

To identify and compare interaction partners of Hsp17, Sip1, and Hsp16.2, Co-IPs were performed; for each condition, at least 3 independent samples were prepared. Experiments were carried out in Protein LoBind tubes from Eppendorf. For each reaction, a 200 μl volume was used with a protein concentration of the *C. elegans* N2 lysate of 1 mg/ml and added purified sHsp had a concentration of 10 μg/ml in PBS + 0.1% NP-40. In the controls, sHsps were left out. The sHsps were added in order to stay consistent with previous literature (24, 50) and to ensure specificity of the Co-IP samples because of the high sequence identities among the *C. elegans* sHsps. Samples were first preincubated for 1 h at 4 °C and then shifted to 20 °C or 37 °C for 45 min. Before the incubation was continued at 4 °C overnight, to each sample, 5 μl antisera and 20 μl Protein G Sepharose Beads (GE Healthcare) were added. Beads were washed twice with 750 μl PBS + 0.1% NP-40 in advance. Purified Hsp17 was used to make the antiserum against Hsp17 (Pineda). The Sip1 antiserum was previously described (24). For Hsp16.2, an antiserum that was made with purified Hsp16.48 (Pineda) was used, which binds Hsp16.2 as well due to the high sequence identity. In the control sample, 5 μl pre-immune serum were added. Samples were incubated overnight at 4 °C while being slowly rotated. The co-IP samples were washed three times with 750 μl PBS + 0.2% NP-40 and a fourth, final washing step was done with 750 μl PBS. All centrifugation steps in between were carried out at 2000 rpm for 2 min at 6 °C. The supernatant was carefully removed, and the bead fractions were stored at -80 °C until the on-bead digestion and desalting step were performed.

### On-bead digestion and sample desalting

After thawing the Co-IP samples, proteins were digested similarly to the protocol from (105). Beads were resuspended in 25 μl buffer I (50 mM Tris pH 7.5, 5 ng/μl trypsin, 2 M urea, 1 mM DTT) and incubated at room temperature. After 2 h, 100 μl buffer II (50 mM Tris pH 7.5, 2 M urea, 5 mM iodoacetamide (IAA)) was added. The samples were further incubated overnight, shaking on a table sample shaker at 25 °C. Then the temperature was switched to 37 °C and incubation was continued for 3 h. 1.5 μl formic acid was added to stop the tryptic digestion. To spin down the Sepharose beads, the samples were centrifuged for 2 min at 510*g*. Afterward, samples were desalted using self-packed stage tips (106). Stage tips were prepared by cutting out small round pieces of a double layer of Empore C18 material (3M) with a diameter of 1 mm and transferring it into the tip. The self-packed stage tips were pre-equilibrated with 70 μl of methanol and prewashed three times with 70 μl 0.5% formic acid with centrifugation step at 960 × *g* in between. Next, 150 μl supernatant from the on-bead–digested samples were added. Samples were again centrifuged and washed three times with 70 μl 0.5% formic acid. As final step, the bound peptides were eluted by adding two times 30 μl buffer III (80% acetonitrile, 0.5% formic acid). Samples were dried after the elution in a speed vacuum concentrator (Eppendorf) and stored at −80 °C. On the day of the measurement, samples were dissolved in 25 μl 1% formic acid, sonicated for 15 min, and then filtered through a Ultrafree PVDF membrane (0.22 μM, Merck Millipore) by centrifugation for 2 min at 7000*g*. Finally, the samples were transferred into Chromacol vials (Thermo Fisher Scientific).

### Tryptic digestion and desalting of the insoluble and soluble fraction

For MS analysis, insoluble fractions were resuspended in 100 μl denaturation buffer (50 mM Tris pH 7.5, 7 M Urea), incubated for 30 min at 37 °C, and thereafter centrifuged for 10 min at 16,900*g*. The supernatant was used for further sample preparation. Soluble fractions were not further pretreated. Proteins were precipitated by the addition of 600 μl acetone and samples were incubated at −80 °C overnight. The next day, the samples were centrifuged for 15 min at 16,900*g*. Protein pellets were washed twice with 800 μl precooled methanol and once sonicated for 10 s. Samples were centrifuged again and the supernatant was completely removed. The pellets were dissolved in 198 μl denaturation buffer and 2 μl 500 mM TCEP were added, followed by an incubation step at 37 °C for 1 h. For alkylation, 4 μl 500 mM IAA were added and the samples were incubated for 30 min at room temperature (RT) in the dark. The reaction was quenched by the addition of 4 μl 500 mM DTT to each sample and followed by another incubation step for 30 min at RT. Subsequently, 600 μl 50 mM Triethylammonium bicarbonate were added as well as 2 μl 500 ng/μl trypsin (Promega), and samples were incubated at 37 °C overnight. The tryptic digest was stopped by the addition of 10 μl formic acid (FA) on the next day. The samples were desalted with 50 mg SEP PAK (tC18) columns (Waters). In advance, the columns were equilibrated with 1 ml acetonitrile, 1 ml elution buffer (80% (v/v) acetonitrile 0.5% (v/v) FA), and three times with 1 ml 0.1% (v/v) TFA. Samples were loaded and the columns were washed three times with 1 ml 0.1% (v/v) TFA and 250 μl 0.5% (v/v) FA. The peptides were eluted three times with 250 μl elution buffer, the first two times by gravity flow, and last time with vacuum applied. The collected samples were dried in a speed vacuum centrifuge (Eppendorf). Afterward, samples were dissolved in 40 μl 1% FA and incubated for 5 min in an ultrasonic bath at RT. The samples were filtered through 0.22 μm centrifugal filters (Merck; 2 min at 16,900*g*). The solutions were transferred into Chromacol vials (Thermo Fisher Scientific) for the measurements.

### MS/MS measurement

MS measurements were carried out on an Orbitrap Fusion that is coupled to an Ultimate3000 Nano-HPLC via an electrospray easy source (Thermo Fisher Scientific). Data were acquired using Xcalibur software (version 3.0sp2, Thermo Fisher Scientific). Peptides were loaded on a 2 cm PepMap RSLC C18 trap column (particles 3 μm, 100A, inner diameter 75 μm, Thermo Fisher Scientific) with 0.1% TFA. Next, for separation, a 50 cm PepMap RSLC C18 column (particles 2 μm, 100A, inner diameter 75 μm, Thermo Fisher Scientific) at 40 °C was used. Each measurement was done with a flow rate of 400 nl/min and a gradient over 135 min, 5 to 90% ACN, 0.1% FA (7 min 5% ACN, 105 min to 22%, 10 min to 32%, 10 min to 90%, 10 min wash at 90%, 10 min equilibration at 5%). Survey scans (m/z 300–1500) were acquired with a resolution of 120,000 and the maximum injection time set to 50 ms (AGC target 2.0e5). The HCD collision energy was set to 30% and charge states 2 to 7 were selected. The maximum injection time in the ion trap was set to 100 ms with an AGC target of 1.0e4. At all available parallelization, time injection of ions was allowed and dynamic exclusion of sequenced peptides was set to 1 min. Fluoroanthene ions (internally generated) were used for real-time mass calibration.

### Analysis of MS/MS data

Obtained MS raw data were analyzed with MaxQuant (version 1.6.2.6) (107, 108). Raw files were searched against the unreviewed *C. elegans* (taxon identifier: 6239) proteome dataset which was downloaded from UniprotDB (date: 20.01.2021). Basically, default settings were applied. All files were assigned to the same fraction and the tryptic cleavage sites before proline were included. For the peptides, up to two missed cleavage sites were allowed as well as a peptide tolerance of 4.5 ppm. Carbamidomethylation was selected as fixed modification, as well as N-terminal acetylation and methionine oxidation were selected as variable modifications. Further, up to 5 modifications per peptide were allowed and the min. ratio count for label-free quantification was set to 1. Peptide length had to be in a range between 7 and 25 amino acids, borders are included. Match between runs was applied (match time window 0.7 min; align window 20 min) and all proteins were identified with a false discovery rate of 1%. Unique and razor peptides were included with a min. ratio count of 1. For the statistical validation of the results, a search against reverse decoy database had been performed. Evaluation of the processed MS was done with Perseus (version 1.6.2.1) (109). The generated datasets of protein groups were by potential contaminant hits, hits from the reverse database, and hits only identified by site. Label-free quantification intensities were log_2_ transformed, grouped into replicates, and the rows filtered on 3 valid values in at least one replicate group. For Hsp17 (20 °C Co-IP sample), 6 samples were prepared which allowed us to filter for 5 valid values in at least one group in Perseus. For the analysis of the insoluble and soluble fractions, rows were filtered on 4 valid values in at least one replicate group. Missing values were calculated from the Gaussian distribution (width: 0.3; downshift: 1.8) and volcano plots with a two-sided *t* test (false discovery rate: 0.05; S_0_ = 0.1) were plotted (110). For detailed analysis of the interactome, only hits with a log_2_ fold change ≥ 2 and a *p*-value ≤ 0.05 were considered. Calculation of molecular weight and pI were done with the ‘compute pI/Mw’ tool on Expasy (87, 111). Hydropathy value was determined with the GRAVY calculator by Stephan Fuchs (http://www.gravy-calculator.de/). Analysis for statistical overrepresentation test of PANTHER Go Slim annotations was performed with the PANTHER online tool (112).

### Crosslinks—sample preparation

Crosslinking reactions were carried out based on a recent published protocol (49) with slight modifications. Each sample, containing 20 μg Hsp17 in 40 μl volume, was preincubated for 1 h at 20 °C. To start the reaction, 4 μl of freshly solubilized (DMSO) 30 mM disuccinimidyl glutarate (Thermo Fisher Scientific) was added. The incubation was continued for 1 h at 20 °C. Subsequently, the reaction was stopped by the addition of 6 μl 1 M Tris (pH 7.4). Next, 29.5 μl denaturation buffer (50 mM Tris; pH 7.4, 6 M Urea) and 2.7 μl 100 mM DTT were added to the samples, followed by a 30 min incubation step at 56 °C to denature the crosslinked proteins. After cooling the samples back to RT, 6.3 μl 100 mM IAA was added and the samples were incubated for 20 min in the dark. For digestion, 1 μl trypsin (sequencing grade 500 ng; Promega) was added and the samples were incubated at 37 °C overnight. To stop the digestion, 1 μl FA was added, samples were desalted, and MS/MS measurements were performed as described for the Co-IP samples.

### Crosslinking—analysis

Obtained raw files were converted with ProteoWizard (version 3.0.21278) (113) into mzML files, which were needed for further evaluation with Kojak (version 2.0.0 alpha2) (114). As database for the analysis, the protein sequence as well as the reverse sequence (as decoy sequence) were provided. The following parameters were set: up to 2 missed cleavages were accepted, the maximum peptide mass was set to 8000 Da, and the minimum was set to 500 Da. The mass tolerance of the precursor was set to 1 ppm. Obtained files were transferred into XML files which are suitable for ProXL (online tool) (115) visualization. Data from different attempts were combined, the minimum score (Kojak) was set to 2.0, and minimum peptide spectrum match value was set to 1.

## Data availability

The accession numbers for the structural data reported in this paper are PDB: 7PE3 and EMDB: EMD-13346. The mass spectrometry proteomics data have been deposited to the ProteomeXchange Consortium via the PRIDE (116) partner repository with the dataset identifiers PXD028080 (reviewer_pxd028080@ebi.ac.uk; Co-IPs), PXD030504 (reviewer_pxd030504@ebi.ac.uk; crosslinks), and PXD038019 (reviewer_pxd038019@ebi.ac.uk; insoluble and soluble fractions).

## Supporting information

This article contains supporting information (56).

## Conflict of interest

The authors declare that there is no conflict of interest.
