# Supplementary material for: The permanently chaperone-active small heat shock protein Hsp17 from Caenorhabditis elegans exhibits topological separation of its N-terminal regions
Source: J Biol Chem. 2022 Nov 26;299(1):102753. doi: 10.1016/j.jbc.2022.102753 (PMC9800568; doi:10.1016/j.jbc.2022.102753)
Supplement: Supplemental Table and Figures [file mmc1.pdf]

Supplementary figure 1: Hsp17::GFP expression pattern in (A) an adult hermaphrodite and (B) a male worm. Detailed fluorescent images showing nematodes in embryonic stages (D-E), the different larval stages L1 (F), L2 (G-I), L3 (J, K) and L4 (L, M) as well as adult (N-P), old adult worms (Q, R), and adult male worms (S-U). White arrows in A-E, I-M, O, P, R-U indicate the excretory cell, canal or associated varicosities. In A, B, F-H and N the pharynx is indicated by yellow arrows. Fluorescence in the intestine is highlighted by orange arrows in A, B, H, J and L. Red arrows in I, K and P indicate Hsp17::GFP expression around the anus. Fluorescence signal around the vulva is indicted in O and Q by blue arrows. In male worms Hsp17::GFP expression at the tail is highlighted by black arrows in S and U. The scale bar shown in U indicates 25 µm and is valid for C to U.

Supplementary figure 2: Percent identity matrix of *C. elegans* sHsps. To highlight similarity within the two main sHsps families a green box is drawn around the identities within the Hsp12 family and a yellow one around the Hsp16 family including Hsp16-related proteins (Sip1, F08H9.3 and F08H9.4). The two splice variants of Hsp17 (cyan box) show a 100 % identity since Q20660 has only one additional amino acid. Identities for Hsp17 towards other *C. elegans* sHsps vary between 19.67 % and 33.33 %.

Supplementary figure 3: Representative negative stain EM image of Hsp17 (A) compared to a typical negative stain EM image of Hsp17ΔNTR (C). (A and C) Scale bars indicate 50 nm. (B) shows the size distribution of Hsp17 with a mean diameter of 12.65 nm (14 875 particles measured). (D) Calibration markers of the SEC-HPLC measurements, which were used to calculate the molecular mass of the Hsp17 and Hsp17ΔNTR oligomers. Additionally, the retention times and the estimated molecular masses of the two investigated proteins are indicated. (E) Native PAGE of Hsp17ΔNTR treated at different temperatures for 30 min (n=3).

Supplementary figure 4: (A-C) HDX-MS measurements of Hsp17 and Hsp17ΔNTR analyzed in comparison. (A) shows the peptide coverage, which was used as basis for the analysis of Hsp17 (B) and its N-terminal deleted variant (C) in comparison. (D and E) HDX-MS measurements and analysis of Hsp17 full-length protein alone. (D) peptide coverage map and (E) HD exchange at 5 different time points (n=3 technical replicates; measured twice; error bars show SD).

Supplementary figure 5: (A) Pearson correlation of the Co-IP results and (B) Analysis of the pI distribution in 0.25 steps of the Hsp17 interactome (blue) and the whole *C. elegans* proteome (fawn-coloured). (C) Hydrophobicity (GRAVY score), rhombuses indicate outliners and stars were used for the mean value in the box plots. (D) Analysis of enriched PANTHER GO-Slim annotations for biological processes. Only processes with a p-value < 0.0005 and a fold enrichment > 4 are shown.

Supplementary figure 6: (A) *In vitro* aggregation assay performed with *C. elegans* lysates and different amounts of Hsp16.2. Samples were separated into soluble and insoluble

fractions after incubation at 37 °C (n=3). (B) Quantification of the insoluble fractions and comparison of the lysate aggregation assays performed with Hsp17 and Hsp16.2 (n=3; error bars show SD). Chaperone activity of Hsp16.2 in the presence of insulin, assays were measured at (C) 20 °C and (D) 37 °C (n=3; error bars show SD).

Supplementary figure 7: MS analysis of the insoluble and soluble fractions of the aggregation assays with *C. elegans* lysate in the presence of Hsp17, Hsp16.2 and Sip1. (A) The number of over- and underrepresented proteins in the soluble and insoluble fraction. Proteins with a $\log_2$ fold change ≥ 2 and a p-value ≤ 0.05 were considered as overrepresented and proteins with a $\log_2$ fold change ≤ 2 and a p-value ≤ 0.05 as underrepresented. (B) Venn diagram of the overrepresented proteins in the insoluble fraction in the presence of the sHsps. (C) Pearson correlation of the analyzed insoluble and soluble fractions (MS samples: n=4).

Supplementary figure 8: Negative stain EM of a time course of chaperone activity assay samples with insulin as substrate at 20°C. The first column shows the substrate only control with 40 µM insulin. Column two and three display chaperone activity assays with 40 µM insulin and Hsp17 concentrations of 12 µM and 24 µM respectively. The fourth column shows Hsp17 only control with a concentration of 24 µM. Row one indicates the starting point before induction of aggregation and rows two to four show samples at 25 min increment. The bottom row shows endpoints of assays after 75 min with 0.25, 1 and 2 µM Hsp17. A selection of these micrographs is shown in Fig. 5. Micrographs were acquired at 10k (upper images) and 60k (lower images) magnification. The red indicated areas show the same part of the sample. Scale bars represent 500 nm and 50 nm respectively.

Supplementary figure 9: Negative stain EM of aggregation assay samples with insulin as substrate at 37°C. The top left micrographs show the substrate only control with 40 µM insulin. Micrographs of the Hsp17 only control are displayed at the bottom right with 8 µM Hsp17. Hsp17 concentrations of the remaining samples are specified and insulin concentration is kept at 40 µM. The micrographs were acquired at 10k (upper images) and 60k (lower images) magnification. The red indicated areas show the same part of the sample. Scale bars represent 500 nm and 50 nm respectively.

Supplementary figure 10: (A) Reconstruction of Hsp17 showing the tetrameric oligomer structure as a local resolution map. Looking at the face of the oligomer (1) one can rotate the model by 54.7° in either the x or y direction to visualize the region where 3 monomers interact (2 and 3). Two consecutive rotations by 45° in x and y direction lead to a view (4) where the dimeric building block of the oligomer with its contacting points to four different dimers can be discerned. The scale bar represents 50 Å. Surface color corresponds to the specified local resolution at this voxel position. (B) Superposition of the Hsp17 reconstruction (blue/orange) and the corresponding AlphaFold prediction (grey, Q20660) (Jumper et al., 2021).

Supplementary figure 11:

Results of the crosslinking experiment with Hsp17 and DSG (n=4), which was used as crosslinker. Data were measured on a Thermo Orbitrap Fusion, analysis was carried out with Kojak and the final visualization was done with Proxl. All lines in (A) indicate cross-links (solid lines) or loop-links (dashed lines). The color of the lines is chosen according to the calculated PSMs (peptide spectrum matches). Hsp17's domain architecture is separated into NTR (lightblue), ACD (darkblue) and CTR (orange), white lines indicate possible crosslink positions, and the obtained coverage is indicated below the scale. In (B-E) the surface monomer is shown in blue while the luminal monomer is orange. The CTRs of neighboring monomers are shown in yellow. (B) Crosslinks (black) between monomers in the dimer. (C) Crosslinks (red) between luminal CTRs and the Hsp17 dimer. (D) Loop-links (purple) in Hsp17. (E) Crosslinks (green) between CTRs of the surface monomers and the Hsp17 dimer. The scale bar shows 10 Å (B-E). (F) Reconstruction of Hsp17 with surface coloring according to possible crosslinks between the NTR of the surface monomer and the remaining structure. Regions depicted in magenta represent the area where the NTR attaches to the ACD in the oligomer. Cyan colored areas show the connecting points of the discovered DSG crosslinks between the NTR and the remaining structure. Orientations according to Sup. Fig. 8A. The scale bar represents 50 Å.

Supplementary figure 12: Visualization of various modes of C-terminal interaction between adjacent dimers. (A) shows the symmetric reconstruction with the fitted part of the model that is involved in the interaction. The density of this part of the oligomer is depicted in red inside the insets. (B) shows asymmetric reconstructions forming three, two and one C-terminal linker of the given type between the building blocks. B1-3 show the links formed by the orange structure motif, and row B4-6 represent the links formed by the blue structure motif. The red densities inside the insets correspond to A and show where missing density is located.

Supplementary table 1: Cryo-EM data collection, refinement and validation statistics

Supplement Figure 1

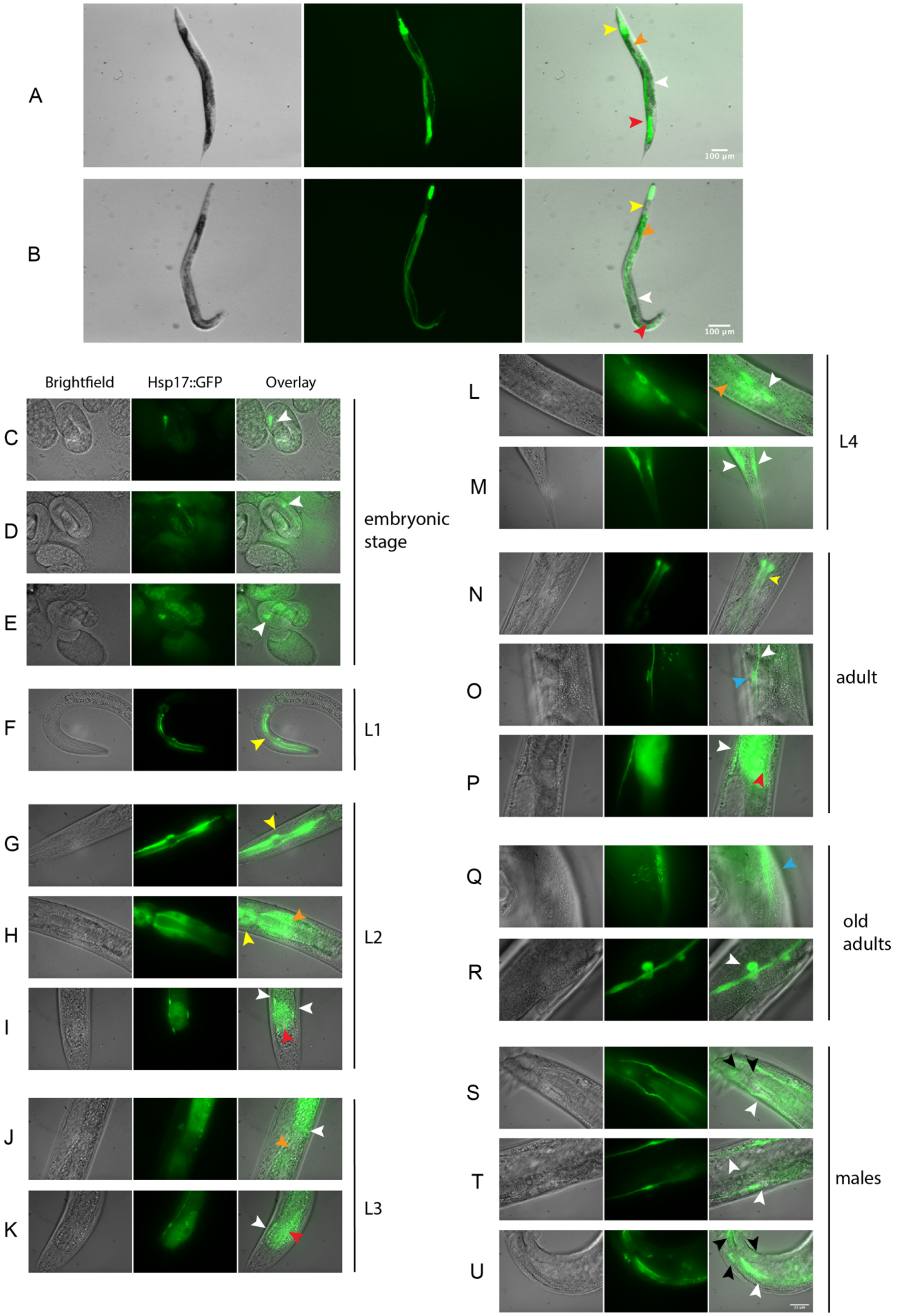

Supplement Figure 2

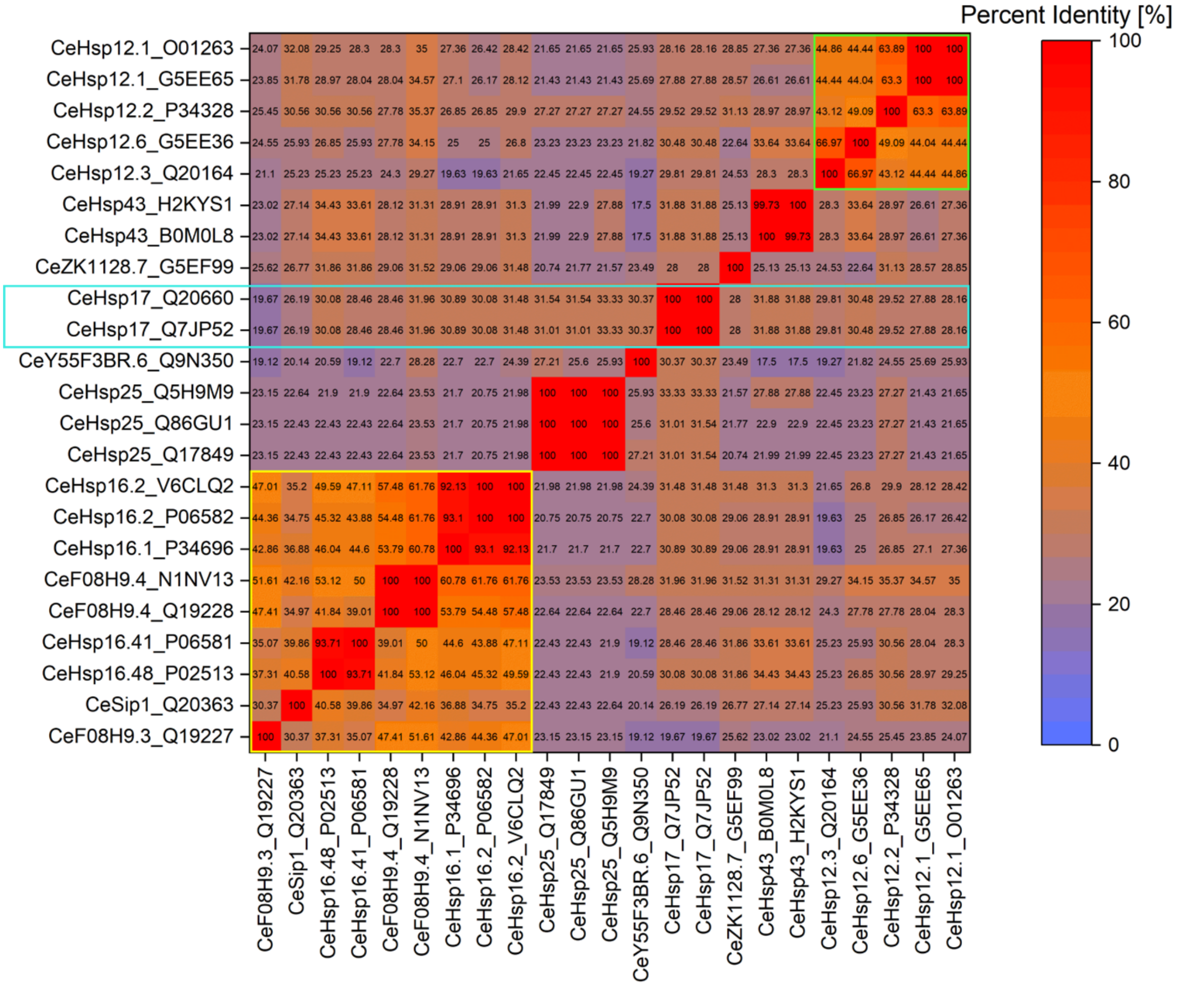

Supplement Figure 3

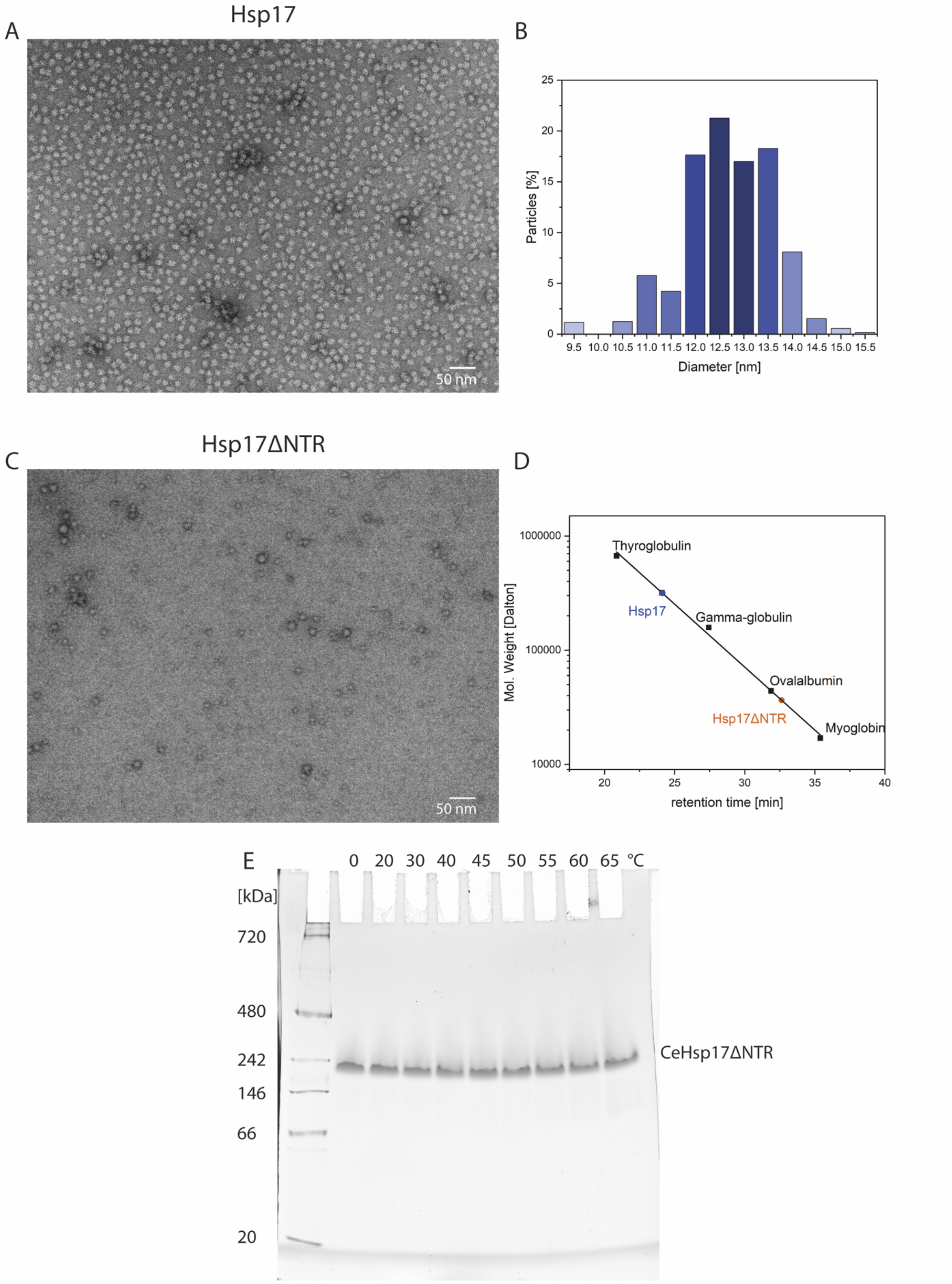

Supplement Figure 4

A

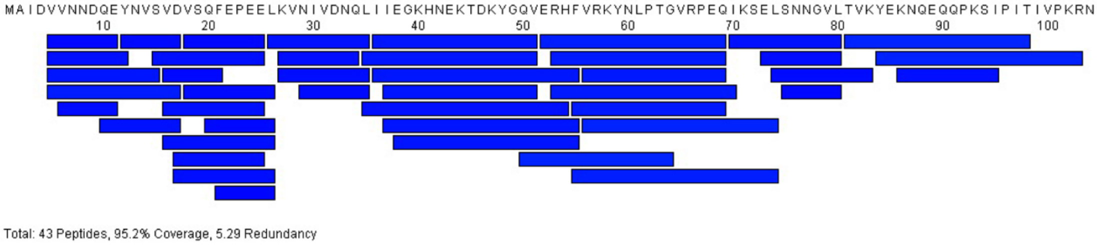

B

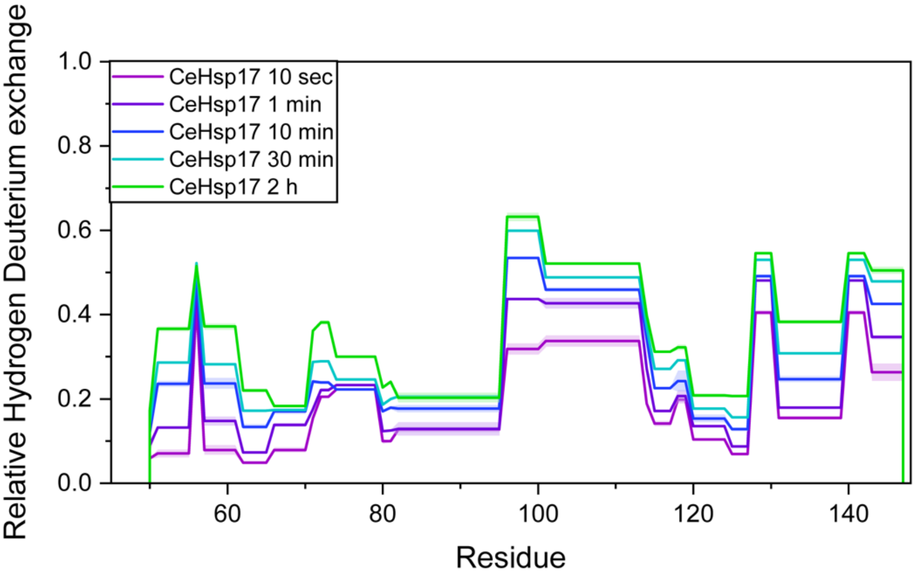

C

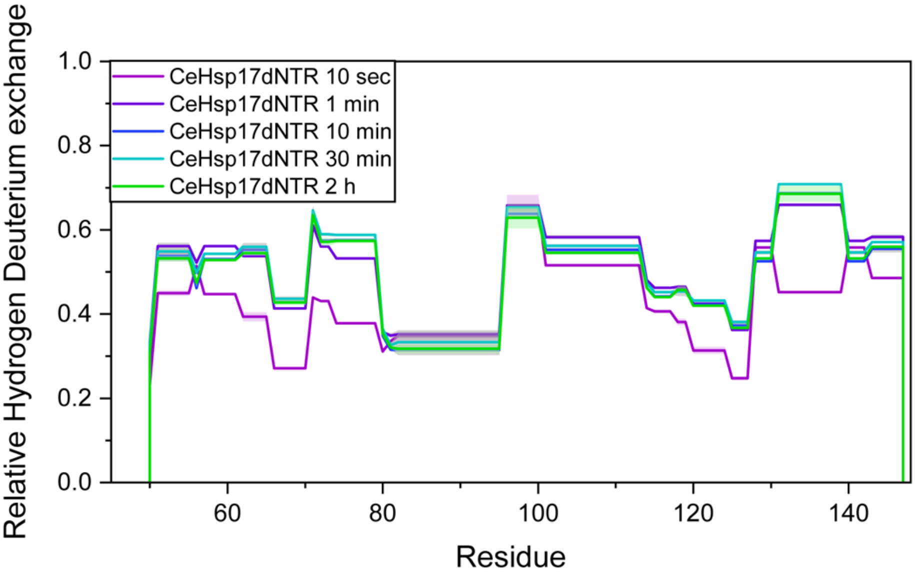

Supplement Figure 4 (continued)

D

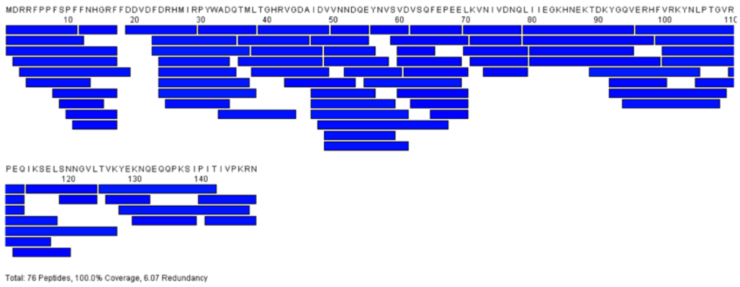

E

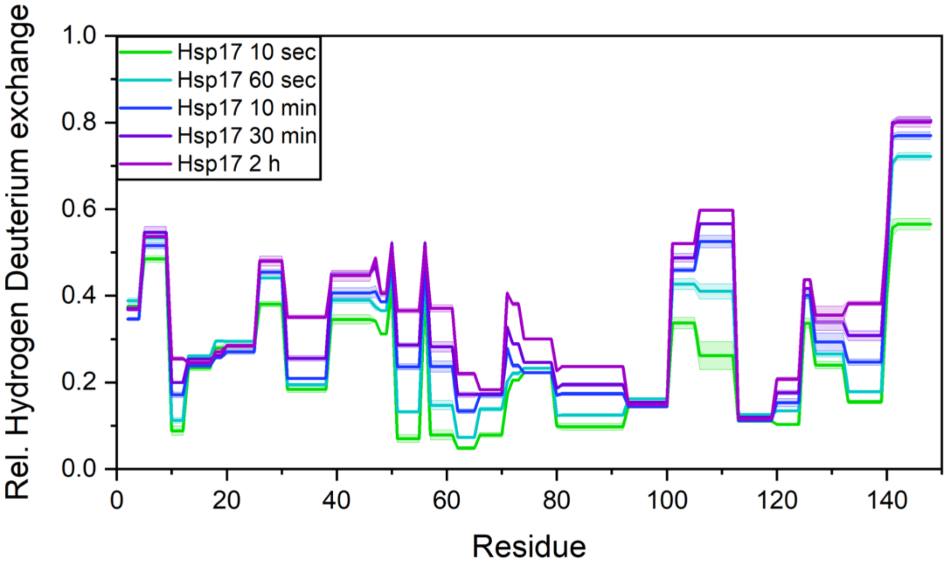

Supplement Figure 5

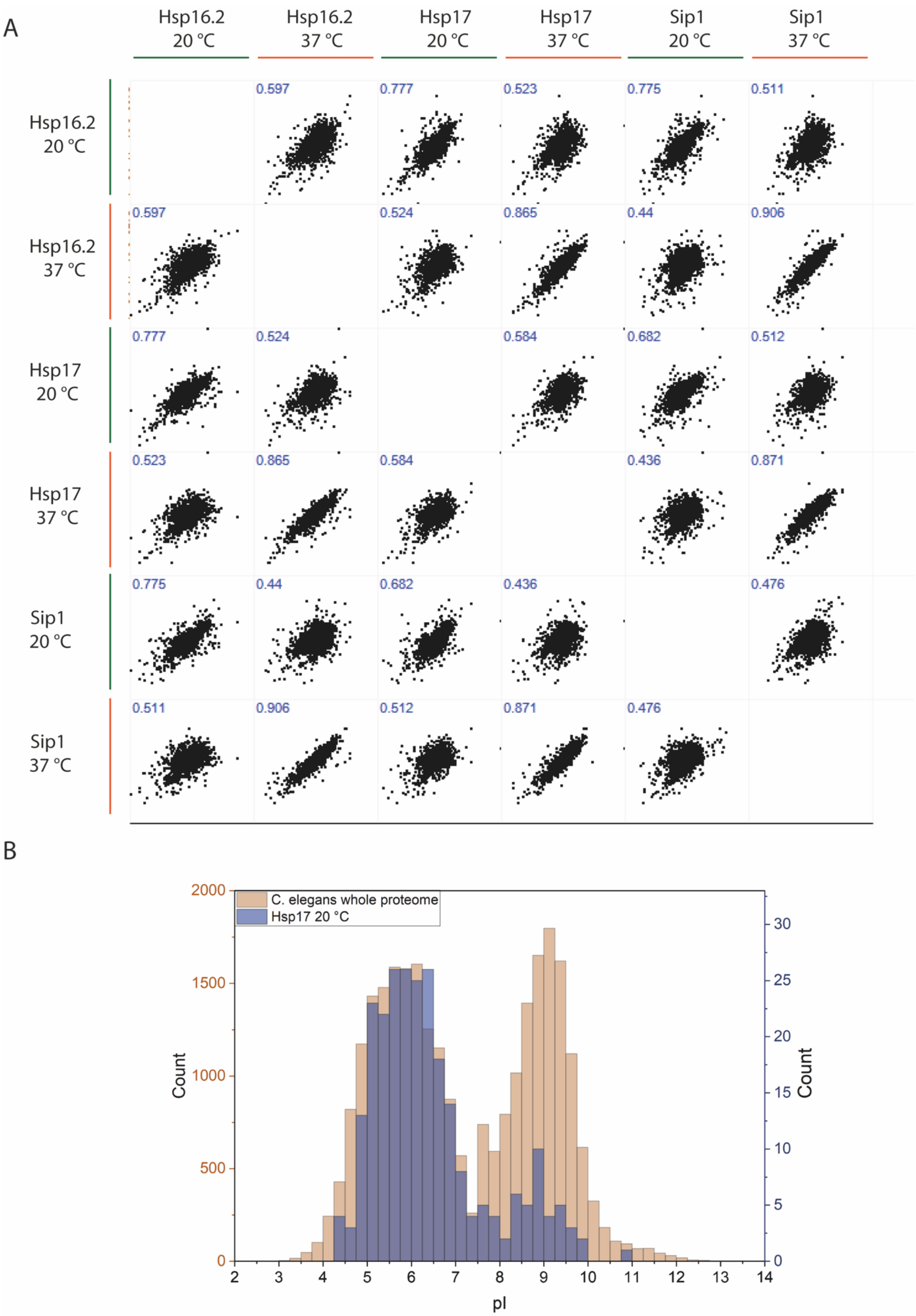

Supplement Figure 5 (continued)

C

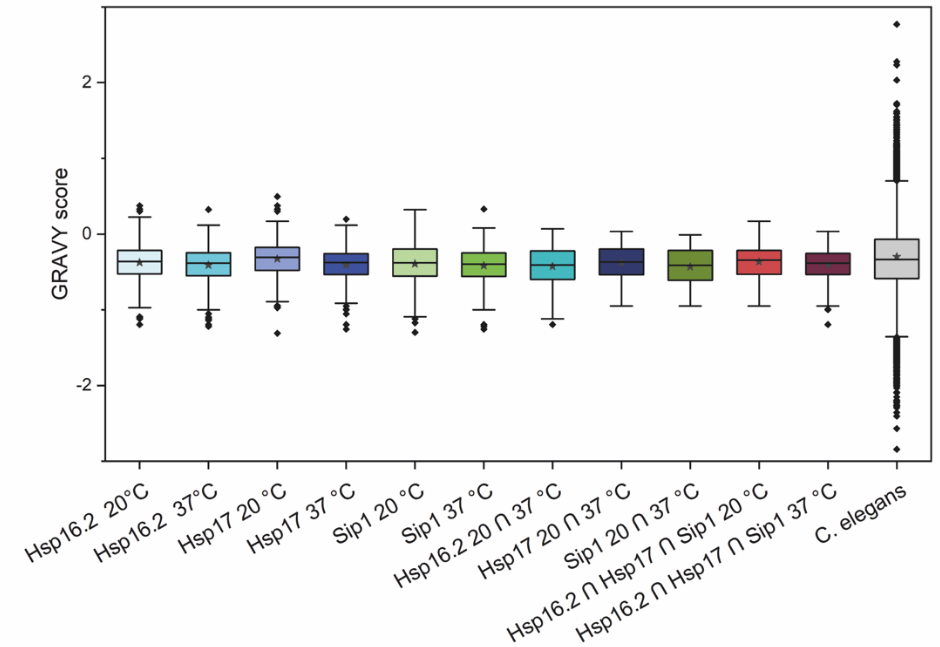

D

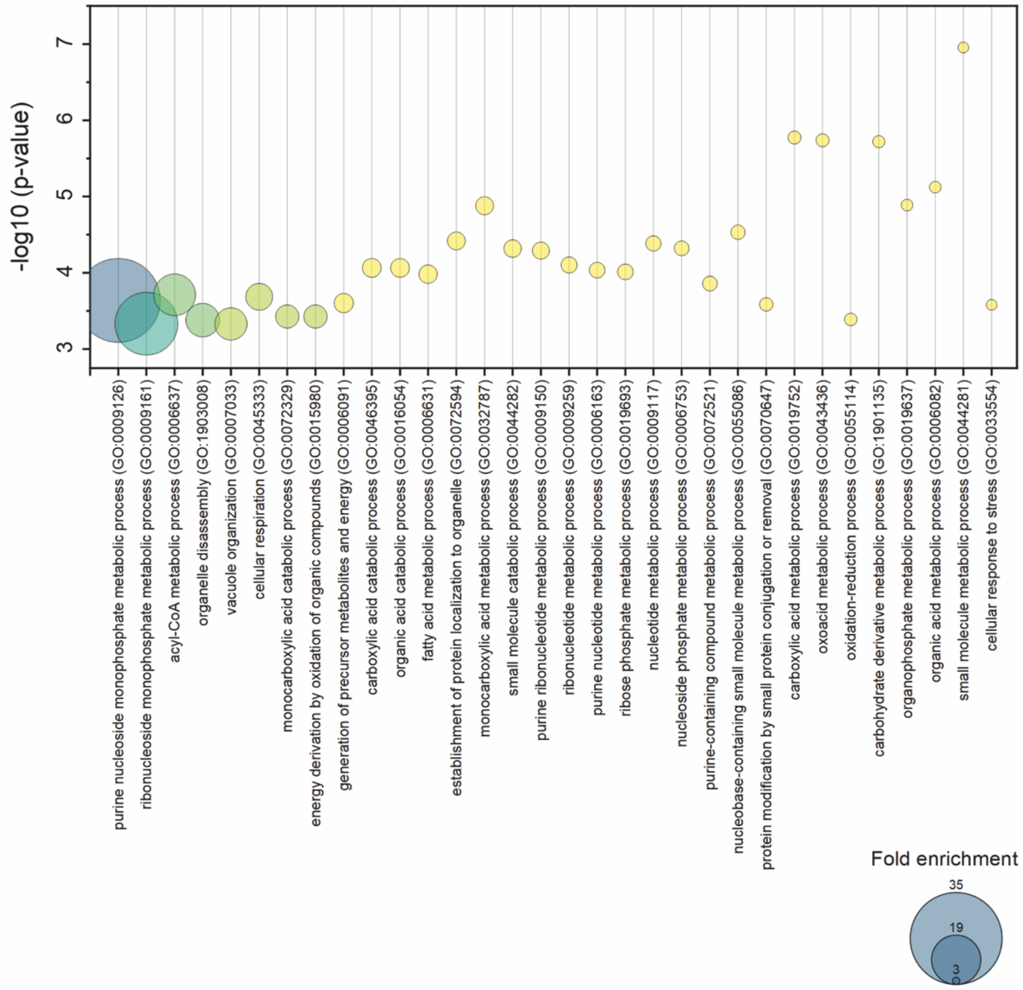

Supplement Figure 6

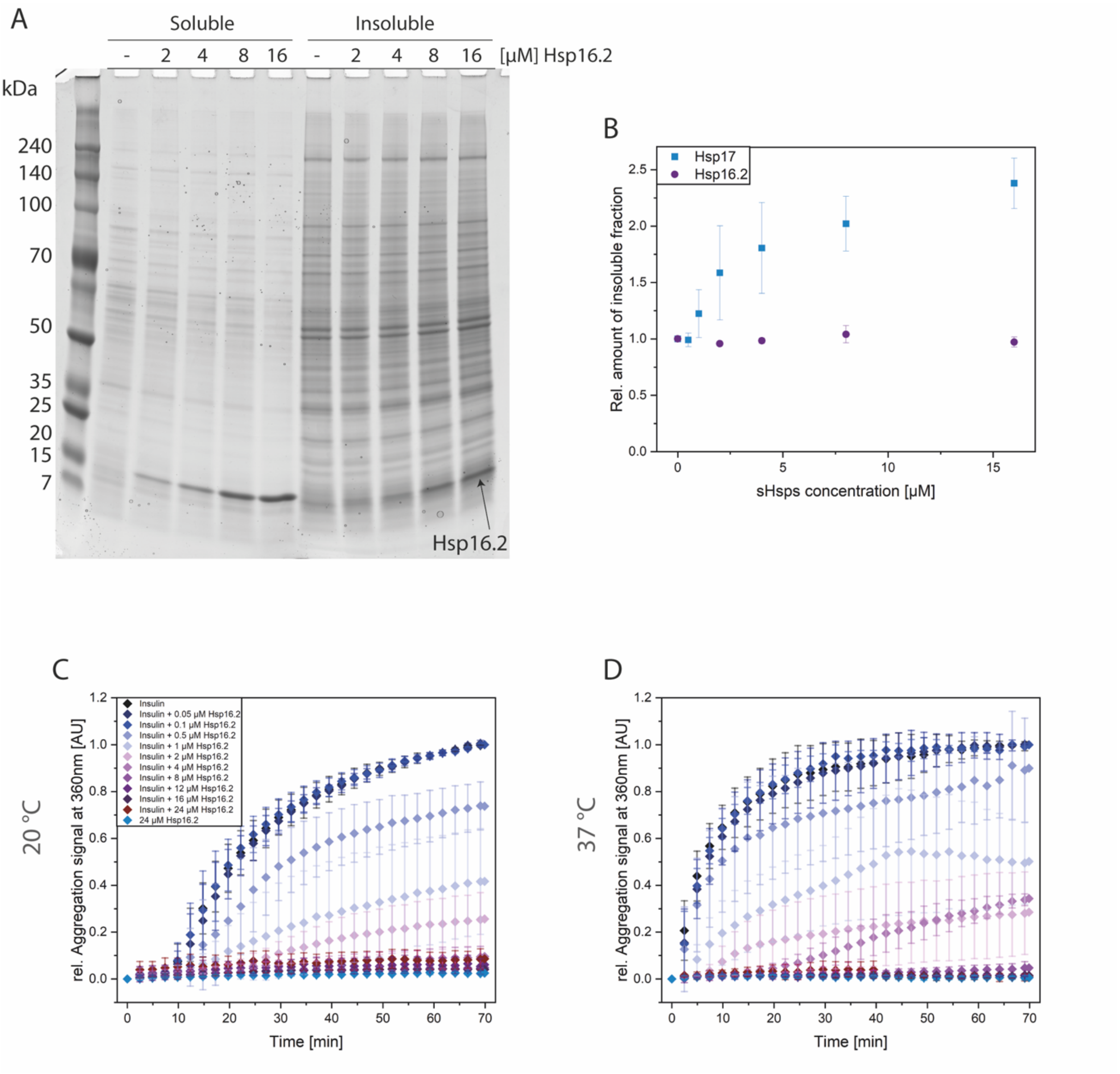

Supplement Figure 7

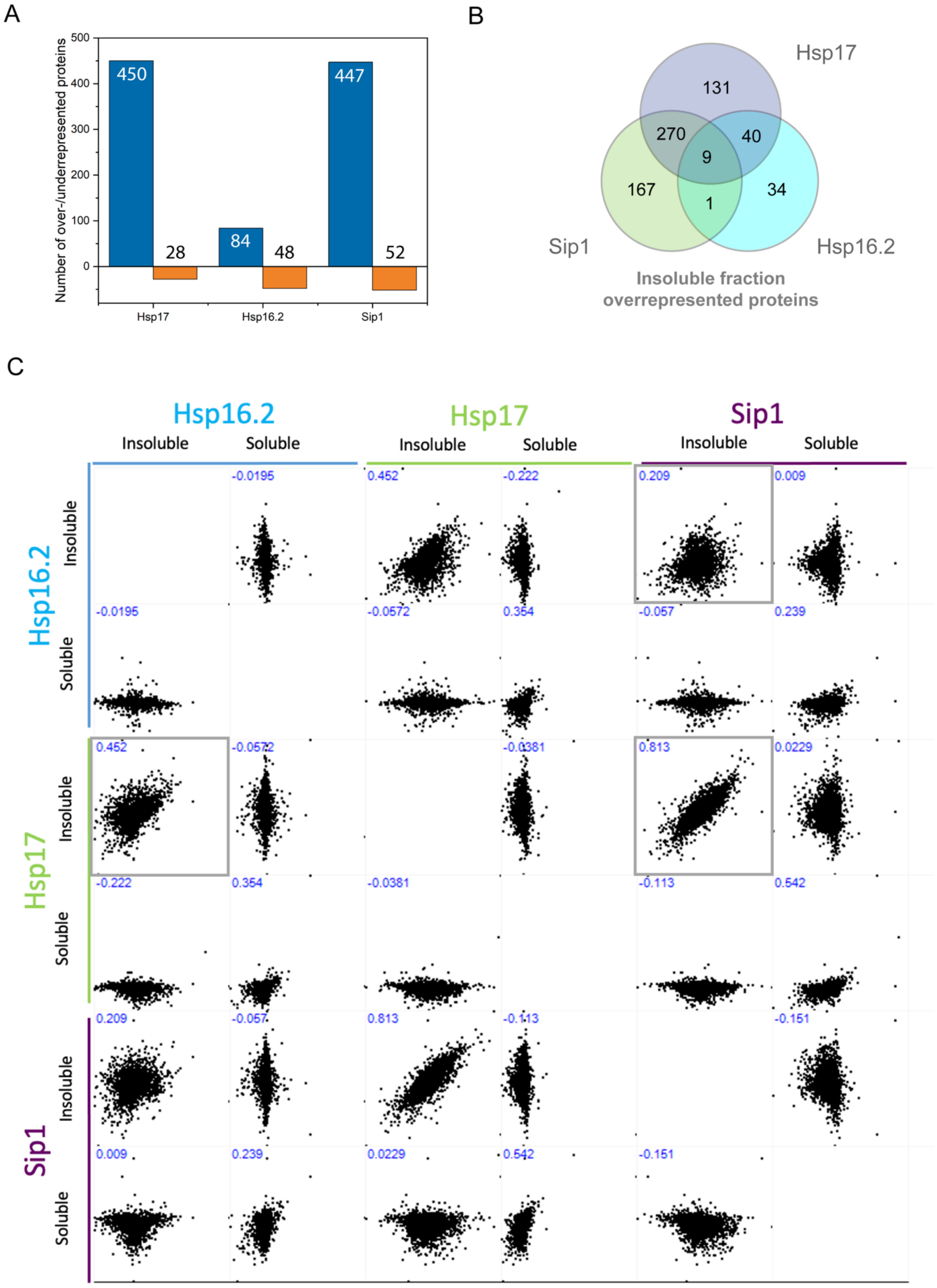

Supplement Figure 8

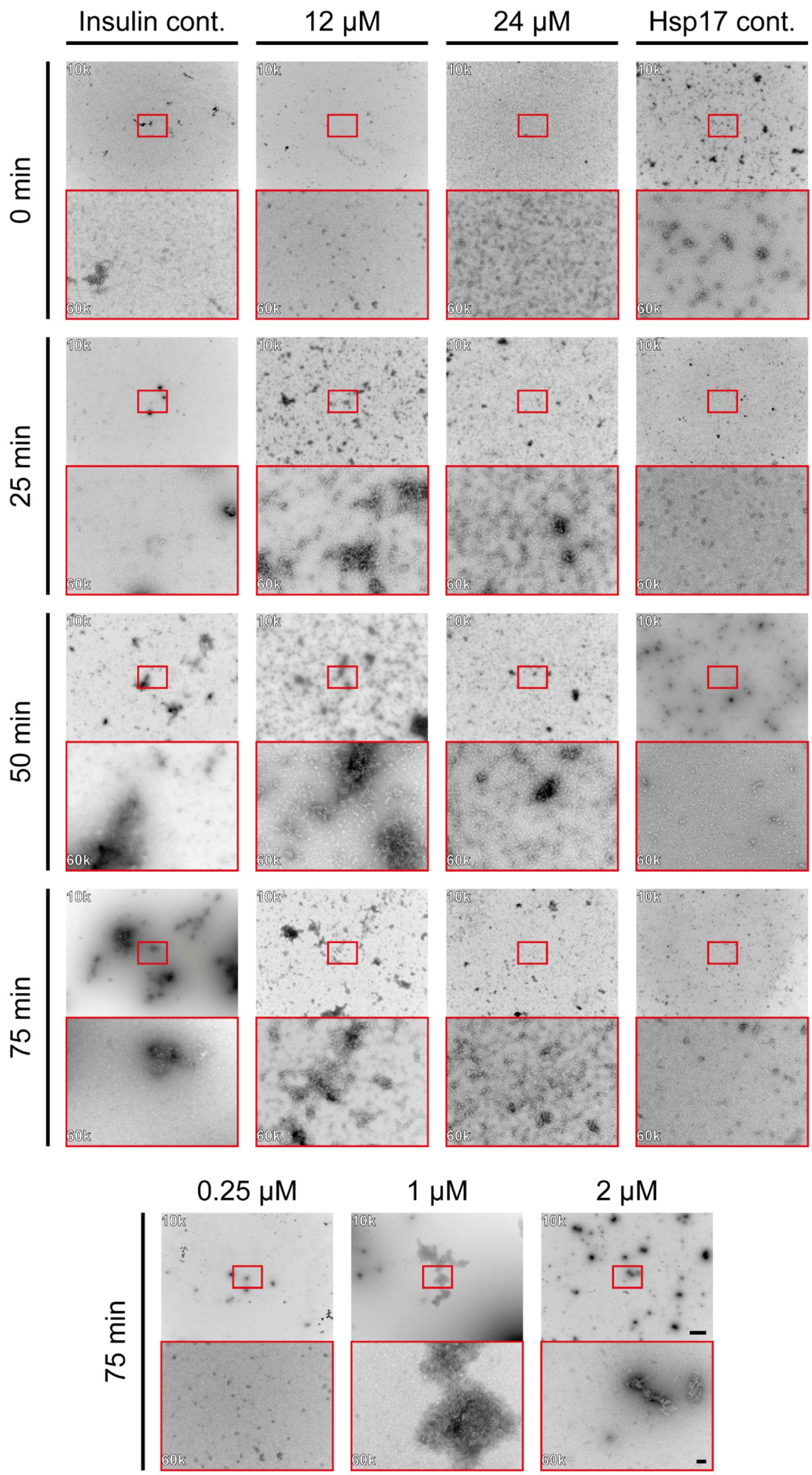

Supplement Figure 9

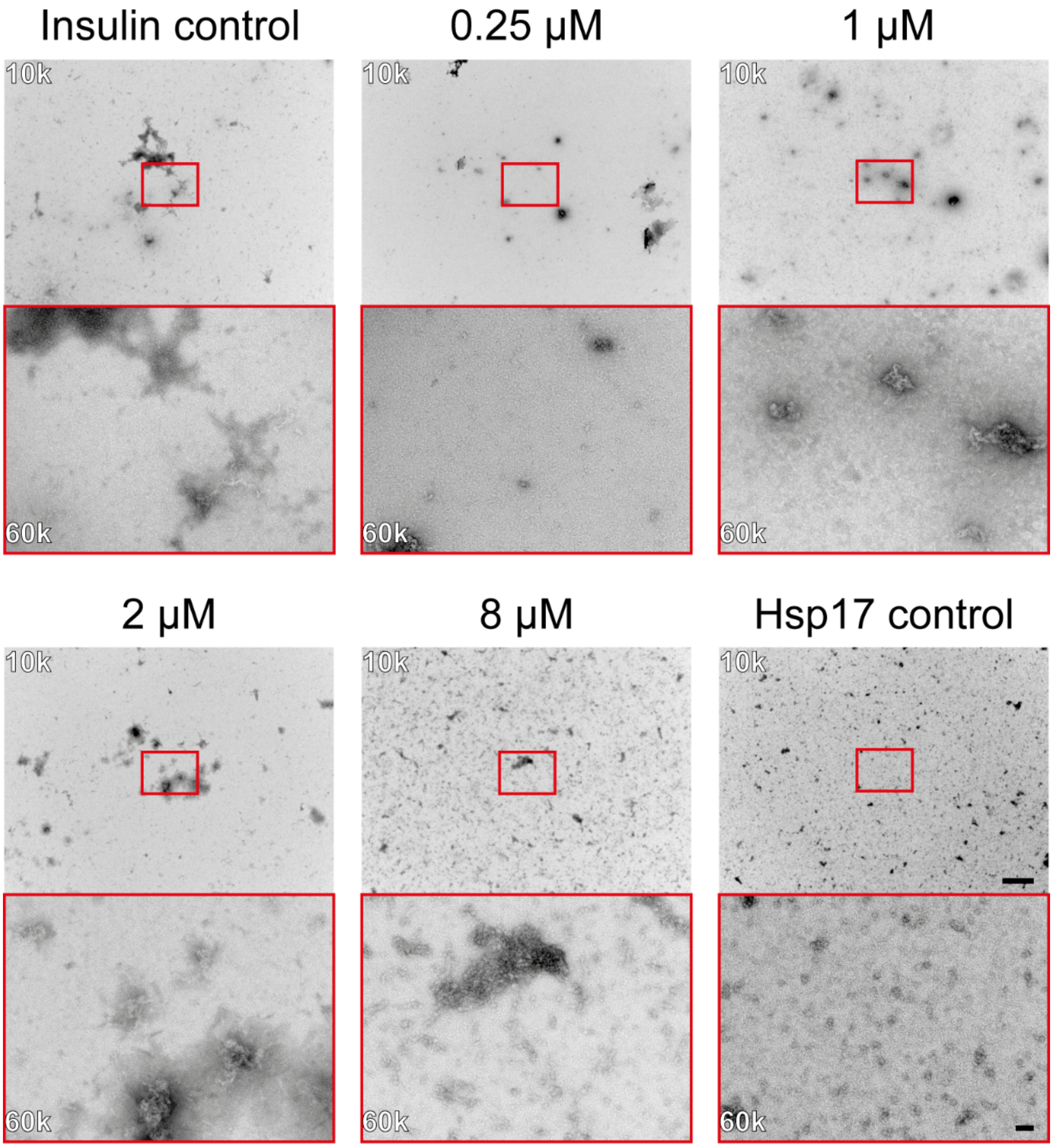

Supplement Figure 10

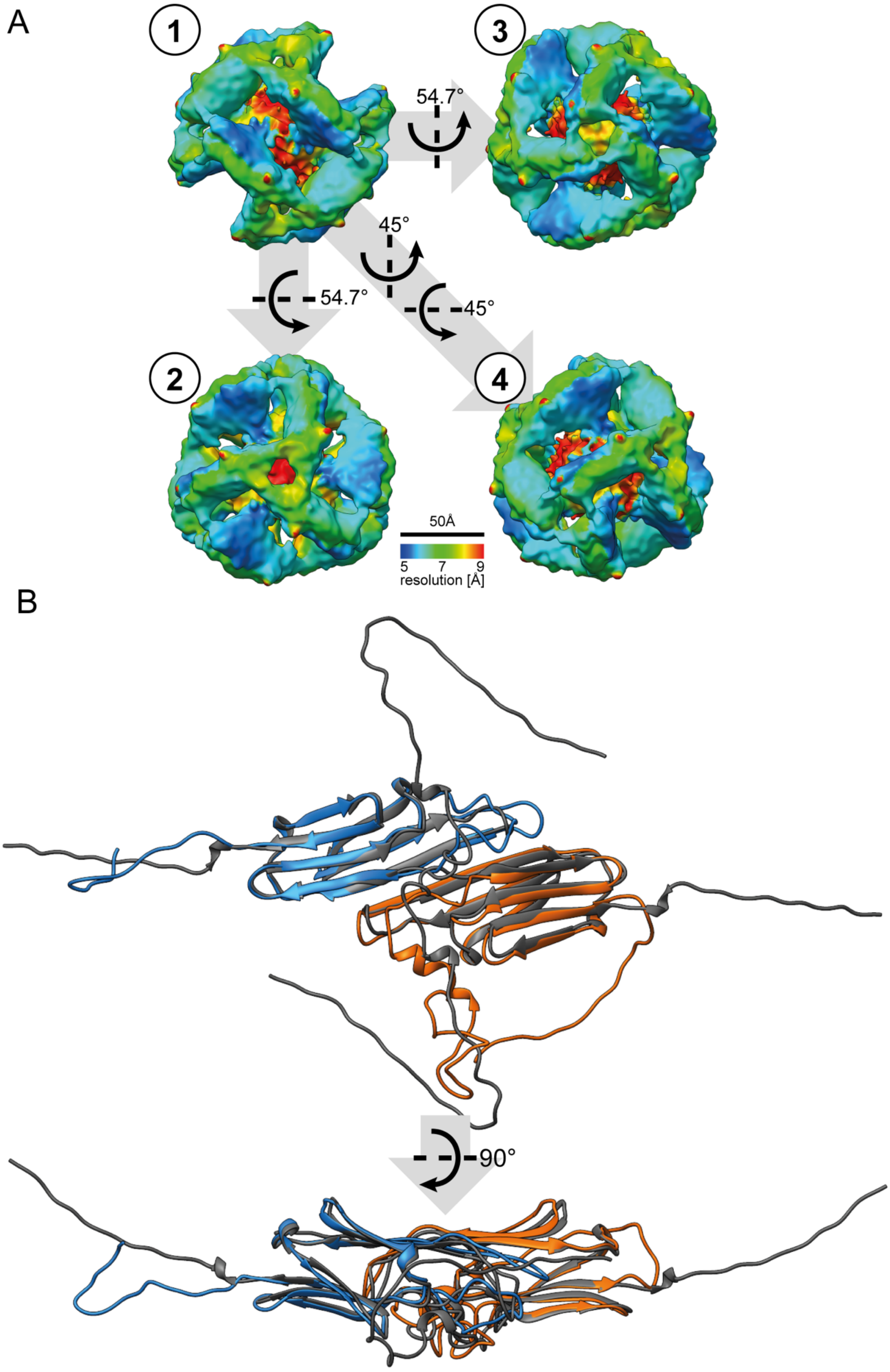

Supplement Figure 11

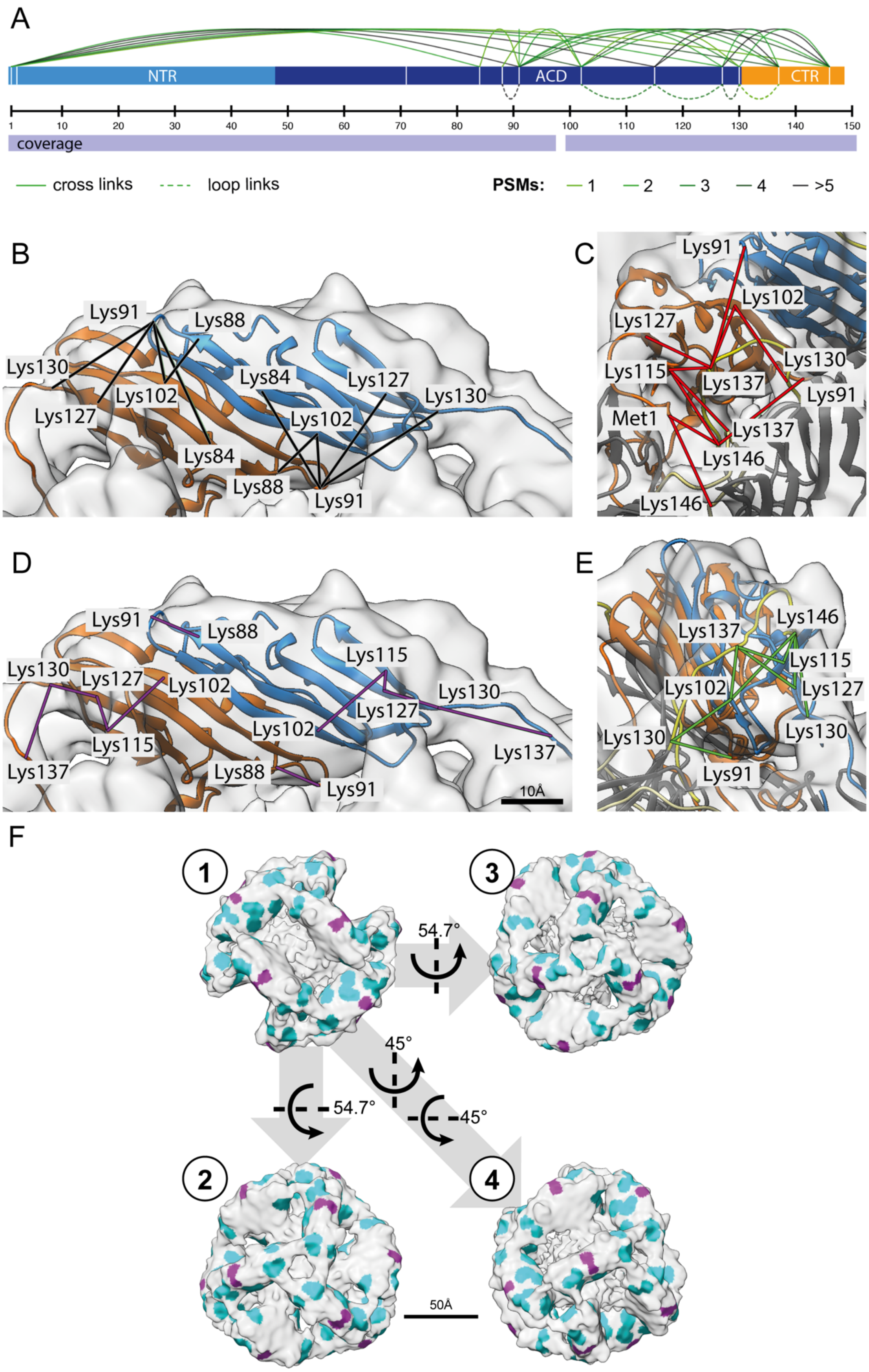

Supplement Figure 12

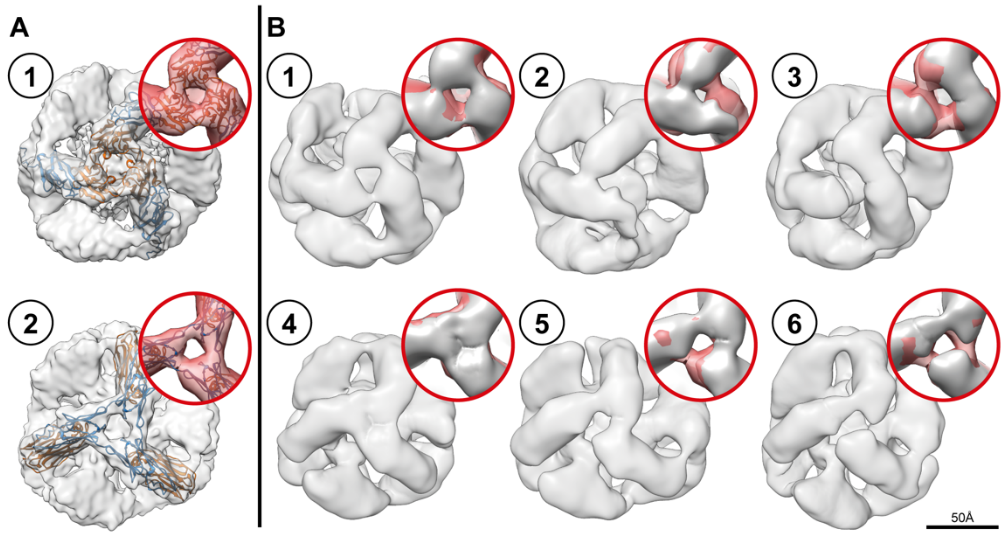

Supplement Table 1

Cryo-EM data collection, refinement and validation statistics

	Hsp17<br>(EMDB- EMD-13346)<br>(PDB 7PE3)
**Data collection and processing**	
Voltage (kV)	300
Electron exposure (e–/Å^2)	55
Defocus range (μm)	-0.5 to -2.5
Pixel size (Å)	1.09
Symmetry imposed	T
Initial particle images (no.)	663141
Final particle images (no.)	187116
Map resolution (Å)	6.49
FSC threshold	0.143
**Refinement**	
Map sharpening *B* factor (Å^2)	494.1
Model composition	
Non-hydrogen atoms	2248
Protein residues	272
R.m.s. deviations	
Bond lengths (Å)	0.004
Bond angles (°)	1.101
Validation	
MolProbity score	2.27
Clashscore	15.51
Poor rotamers (%)	0
Ramachandran plot	
Favored (%)	89.18
Allowed (%)	10.82
Disallowed (%)	0
